# Protective role of lactate in allergic airway inflammation: mitigation of inflammatory injury, epithelial barrier integrity restoration, and gut-lung axis involvement

**DOI:** 10.1186/s12931-026-03806-z

**Published:** 2026-07-13

**Authors:** Meilian Lin, Jie Zhou, Yan Wang, Wenchun Xu, Xiaoyu Sun

**Affiliations:** 1 Key Laboratory of Laboratory Medical Diagnostics Designated by the Ministry of Education, School of Laboratory Medicine, Chongqing Medical University, Chongqing, China; 2 Department of Clinical Laboratory, National Clinical Research Center for Children and Adolescents’ Health and Diseases, Ministry of Education Key Laboratory of Child Development and Disorders, Chongqing Key Laboratory of Pediatric Metabolism and Inflammatory Diseases, Children’s Hospital of Chongqing Medical University, Chongqing, China; 3 Department of Clinical Laboratory, The First Affiliated Hospital of Chongqing Medical University, Chongqing, China; 4 Department of Laboratory Medicine, The Second Affiliated Hospital of Chongqing Medical University, Chongqing, China

**Keywords:** Lactate, Allergic airway inflammation, Airway barrier function, Gut microbiota

## Abstract

**Background:**

Allergic asthma is a prevalent respiratory disorder characterized by chronic airway inflammation and remodeling. Glycolysis has been reported to participate in pathogenesis of allergic asthma and increased lactate levels were found in asthma patients and mouse models. However, the function of lactate in allergic asthma remains unclear.

**Methods:**

A mouse model of HDM induced allergic airway inflammation was established. Six age- and weight-matched female mice were assigned to different groups using a randomized double-blind method. A panel of indicators such as serum IgE, infiltration cell numbers, Th2 cytokines levels and eosinophil extracellular traps (EETs) were applied to assess airway inflammation. Airway epithelial barrier function was measured by Western blot and immunofluorescent staining. RNAseq analysis of lung tissues was applied to elucidate potential mechanisms, and 16S rRNA gene sequencing of fecal samples was used for gut microbiota analysis.

**Results:**

Administration of lactate could significantly ameliorate allergic airway inflammation including Th2 cytokines, inflammatory cell infiltration, histopathological morphological changes together with eosinophil extracellular traps (EETs) formation in a mouse model of asthma. Moreover, RNAseq analysis revealed that lactate decreased proinflammtory cytokine and chemokine related pathways such as MAPK, STAT1, STAT3 and NF-κB to exert immunoregulatory effects. In addition, we found that lactate dramatically inhibited airway epithelial barrier dysfunction and pulmonary apoptosis. Furthermore, 16S rRNA gene sequencing of fecal samples suggested that lactate treatment increased abundance of *Lactobacillus*, *Limosilactobacillus* and *Bacteroides*, showing a shift towards a healthier state in HDM-induced asthmatic mice.

**Conclusions:**

Our study integrating transcriptomic and microbiome analyses, revealed a protective effect of lactate on allergic airway inflammation, providing a basis for development of novel therapeutic treatment for allergic asthma.

**Graphical Abstract:**

**Figure:**
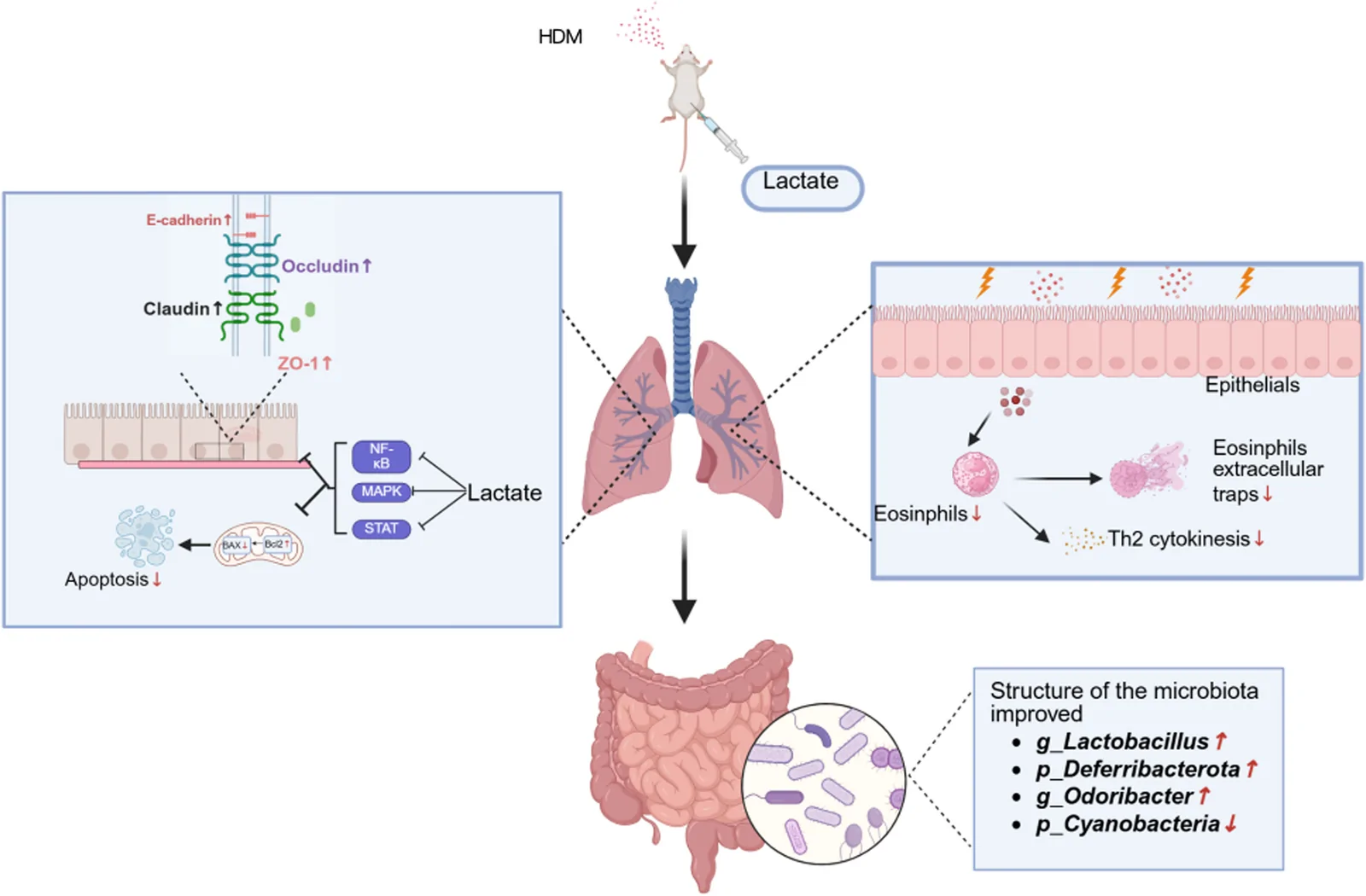
Schematic summary illustrating the protective role of lactate in allergic airway inflammation.

**Supplementary Information:**

The online version contains supplementary material available at https://doi.org/10.1186/s12931-026-03806-z.

## Introduction

Allergic asthma is a chronic inflammatory disease, characterized by airway inflammation, airway hyperresponsiveness (AHR) and airway remodeling. The pathophysiology of allergic asthma is complex and heterogeneous by various host-environment interactions occurring at various scales, triggering a Th2 dominated inflammatory responses. Specifically, Th2 cells along with eosinophils, airway epithelial cells, macrophages and type 2 innate lymphoid cells (ILC2) together with type 2 cytokines including IL-4, IL-5 and IL-13 accelerate allergic airway inflammation [1].This chronic respiratory condition impacts over 300 million people in the worldwide, which poses a huge global health burden. Although traditional pharmacologic strategies has made a progress in asthma control, it remains incurable and it is therefore urgent to develop novel therapeutic approaches to improve patient outcomes [2].

Recent studies have highlighted the role of metabolic disturbances in exacerbating asthma progression including lipid metabolism alterations, amino acid metabolism changes and glycolysis [3]. Importantly, glycolytic reprogramming plays an important role in glucose metabolism, which acts as a bridge between metabolic process and inflammatory responses [4]. Cells can exhibit the glycolysis process even under aerobic conditions to meet their high metabolic demands, which is referred to as “Warburg effect”. It is notable that glycolysis can convert glucose to produce lactate and biomass precursors [5]. Lactate as a glycolytic marker exerts a dual role in different inflammatory conditions. On the one hand, lactate inhibits NOD-like receptor thermal protein domain associated protein 3 (NLRP3) inflammasome by GPR81 pathway, and then reduces pro-inflammatory cytokine IL-1β production to exert anti-inflammatory activity in models of pancreatitis and liver injury [6]. It has been reported that lactate can alleviate inflammatory responses through downregulation NF-κB signaling activation [7]. On the other hand, lactate activates its receptor Slc5a12 expression to drive lactate uptake into CD4^+^ T cells, contributing to production of IL-17 via PKM2/STAT3 signaling in murine model of arthritis [8]. Elevated lactate is observed in septic patients and high lactate levels is linked to mitochondrial damage and respiratory function impaired, leading to the progress of inflammatory responses and multiple organ dysfunction syndromes (MODS) [9]. In asthma, research reveals that glycolysis is closely related to asthma pathogenesis and disease severity [10]. In allergic asthma mouse model, increased pulmonary glycolytic flux, glucose use and expression of glycolytic enzymes are observed [11]. Application of glycolysis inhibitors such as glucose transporter 1 (Glut1), lactate dehydrogenase A (LDHA) and 2-Deoxy-d-glucose (2-DG) significantly attenuates airway inflammation, oxidative stress and pyroptosis [12]. An increased lactate has been discovered in blood, sputum and bronchial epithelial cells of asthma patients [13]. However, precise mechanisms involving elevated lactate in allergic asthma remain unknown.

In our current study, we observed that lactate played an immunoregulatory role in allergic asthma. Lactate suppressed inflammatory cells infiltration, eosinophils extracellular traps formation and type 2 cytokine release as well as airway epithelial cell dysfunction. Moreover, lactate alleviated activation of MAPK, STAT1, STAT3 and NF-κB signaling pathways, and regulated Bax/Bcl-2/Caspase 3 cascades to anti-apoptosis process. In conclusion, our study uncovered the protective role of lactate in allergic asthma and provided a potential therapeutic method for asthma control.

## Materials and methods

### Mouse models and treatment

Female BALB/c mice, aged 6–8 weeks and weighing 18–20 g, were purchased from the Laboratory Animal Center of Chongqing Medical University (Chongqing, China). All animals were housed under specific pathogen-free (SPF) conditions in a temperature-controlled environment (22 ± 2 °C) with a 12-hour light-dark cycle and provided with free access to standard rodent chow and drinking water. All animal experiment procedures adhered to the 3Rs principles (Replacement, Reduction, Refinement), and were approved by the Animal Ethics Committee of Chongqing Medical University (Approval No. IACUC-CQMU-2023-0034). For the HDM-induced airway inflammation model, mice were sensitized by administering 1 µg of HDM (Greer Laboratories, cat#XPB82D3A25) intranasally on Day 0. Subsequently, mice received a sensitization challenge with 10 µg HDM intranasally between Days 7 to 11. The lactate treatment group received 1 mg/kg via intraperitoneal injection 2 h before each challenge. The control group mice was treated intranasally with PBS alone without lactate treatment, mice in the HDM group were injected intraperitoneally with the same volume of vehicle (PBS) instead of lactate, serving as the negative control for the lactate treatment group. Mice were sacrificed within 48 h after the final sensitization, and bronchoalveolar lavage fluid (BALF) and lung tissue samples were collected for subsequent experiments.

### Bronchial alveolar lavage fluid collection

Mice were euthanized by injection of an overdose of sodium pentobarbital 48 h after the final challenge, and BALF was collected. Briefly, after ligating the main bronchi of the mice, a catheter was inserted into the trachea, and BALF was collected by aspirating lung tissue three times with 0.5 ml of pre-cooled sterile PBS. The BALF was centrifuged (1000 g, 5 min, 4℃). The cell pellets were resuspended in 0.5 mL PBS and prepared for cell quantification and morphological analysis using Wright-Giemsa staining (Solarbio). Two researchers independently counted and typed a minimum of 200 cells/sample.

### Serum IgE measurement

After collecting mouse blood samples, let them stand for 30 min. Subsequently, centrifuge at 3000 rpm for 30 min to collect the supernatant. Determine serum IgE concentration using an ELISA kit (Biolegend, Cat #432404) according to the manufacturer’s provided protocol.

### Enzyme-linked immunosorbent assays (ELISA)

Mouse IL-4, IL-5, IL-6 and IL-13 concentrations in lung homogenate were quantified via ELISA assays (mouse IL-4, IL-5, IL-6 and IL-13: Biolegend, Cat # 431104; Biolegend, Cat # 431204; Biolegend, Cat # 431304; R&D Systems, Cat # DY413-05). The assays were meticulously conducted in strict accordance with the manufacturer’s provided protocol. The concentration of ECP-DNA was measured according to the previous study [19].

### Lung tissue pathological examination

Mouse lung tissue samples were fixed in 4% paraformaldehyde solution for 48 h, followed by sequential ethanol dehydration and embedding in paraffin blocks. Continuous 4 μm-thick sections were prepared. Following preparation, tissue sections were subjected to staining with either hematoxylin and eosin (H&E; Solarbio, Cat # G1120) or periodic acid-Schiff (PAS; Beyotime Biotechnology, Cat # C0142M) according to the manufacturer’s provided protocol. Stained sections were observed and photographed under a conventional light microscope for the evaluation of histopathological alterations in the lung tissues. Quantitative scoring was performed for both H&E‑ and PAS‑stained lung tissue sections. For H&E‑stained sections, 0 points indicated no inflammatory cell infiltration around airways; 1 point indicated a small number of inflammatory cells; 2 points indicated a single layer of inflammatory cells; 3 points indicated 2 to 4 layers of inflammatory cell infiltration; 4 points indicated more than 4 layers of inflammatory cell infiltration. For PAS‑stained sections, 0 points indicated no magenta staining in the airway epithelium; 1 point indicated less than 25% of epithelial cells stained positive; 2 points indicated 25–50% of epithelial cells stained positive; 3 points indicated 50–75% of epithelial cells stained positive; 4 points indicated more than 75% of epithelial cells stained positive.

### Immunofluorescence staining

For immunofluorescence staining, tissue sections underwent antigen retrieval via microwave treatment in citrate buffer, followed by blocking with 5% bovine serum albumin (BSA) at room temperature for 1 h. Sections were incubated overnight at 4 °C with primary antibodies, washed three times with PBS, incubated with fluorescently labeled secondary antibodies, and and counterstained with DAPI for nuclear staining. The primary antibodies used in this study were as follows: ECP (proteintech, Cat No. 55338-1-AP), CitH3 (ABclonal, Cat #A18298), Cleaved caspase 3 (Huabio, Cat # ET1602-47), ZO-1 (ProteinTech, Cat # 21773-1-AP), E-cadherin (Cell Signaling Technology, Cat # 3195), Occludin (Huabio, Cat # ET1701-76), Claudin-1 (Huabio, Cat # HA721248). For each experiment, multiple randomly selected microscopic fields were analyzed and averaged to obtain a representative value. Both image acquisition and fluorescence quantification were performed in a blinded manner by investigators unaware of group allocation.

### TUNEL detection

TUNEL staining was performed using an in situ cell death detection kit (Beyotime Biotechnology, Cat # C1088). Three random independent visual fields were imaged per lung section at fixed magnification. The integrated TUNEL fluorescence intensity of each field was quantified with ImageJ software (1.54p) to reflect pulmonary apoptosis. All image acquisition, field selection, and quantitative analysis were conducted in a blinded fashion.

### RNA isolation and quantitative real-time PCR

Total RNA was extracted from lung tissues via the phenol-chloroform extraction method. Purified RNA was reverse-transcribed into complementary DNA (cDNA) using the PrimeScript™ RT reagent Kit (TaKaRa, Cat # RR037A). Quantitative real-time PCR (qRT-PCR) was carried out with SYBR Green Master Mix (Tsingke Biotechnology Co., Ltd., Cat # TSE202) and gene-specific primers. Relative mRNA expression of target genes was quantified by the 2^−ΔΔCt method, with β-actin serving as the housekeeping reference gene. Briefly, ΔCt = Ct_target − Ct_β-actin and ΔΔCt = ΔCt_treatment − ΔCt_control. All gene expression data are presented as fold change normalized to the control group. The full sequences of all primers are summarized in Table 1.

**Table 1:** Primer sequences

Gene	Primer sequence
Mouse IL−4	5’-GGTCTCAACCCCCAGCTAGT − 3’ 3’-GCCGATGATCTCTCTCAAGTGAT-5’
Mouse IL−5	5’-TCAGGGGCTAGACATACTGAAG-3’ 3’-CCAAGGAACTCTTGCAGGTAAT-5’
Mouse IL−6	5’-ACAAAGCCAGAGTCCTTCAGAG-3’ 3’-TCTGTGACTCCAGCTTATCTCTTG-5’
Mouse IL−13	5’-TGAGCAACATCACACAAGACC-3’ 3’-GGCCTTGCGGTTACAGAGG-5’
Mouse Claudin−1	5’-TGGGGCTGATCGCAATCTTT-3’ 3’-CCCAATGACAGCCATCCACA-5’
Mouse Occludin	5’TTGAAAGTCCACCTCCTTACAGA–3’ 3’-CCGGATAAAAAGAGTACGCTGG-5’
Mouse E-cadherin	5’-AACCCAAGCACGTATCAGGG-3’ 3’-GAGTGTTGGGGGCATCATCA-5’
Mouse ZO−1	5’-CTCAAGTTCCTGAAGCCCGT-3’ 3’-GCAAAAGACCAACCGTCAGG-5’
Mouse β-actin	5’-GTGACGTTGACATCCGTAAAGA-3’ 3’-GCCGGACTCATCGTACTCC-5’

### Western bloting

Total proteins were extracted from lung tissues using RIPA lysis buffer (Beyotime Biotechnology, China), and the protein concentrations were determined by the BCA method. After separation by 8%–12% SDS-PAGE, proteins were transferred on to PVDF membranes (Millipore Corp., Billerica, MA, USA). The membranes were blocked with 5% skim milk for 2 h at room temperature and washed three times with TBST. Primary antibodies—ECP (proteintech, Cat No. 55338-1-AP), CitH3 (ABclonal, Cat #A18298 ), ZO-1 (ProteinTech, Cat # 21773-1-AP), E-cadherin (Cell Signaling Technology, Cat # 3195), Occludin (Huabio, Cat # ET1701-76), Claudin-1 (Huabio, Cat # HA721248), Bax (Huabio, Cat # ET1603-34), Bcl-2 (Huabio, Cat # HA723111), Cytochrome C (Huabio, Cat # ET1610-60), Cleaved Caspase 3 ((Huabio, Cat # ET1602-47) were incubated overnight at 4 °C. After washing with TBST incubation with secondary antibodies for 1 h at room temperature. Protein bands were visualized using an ECL kit (ECL-Plus; Beyotime Biotechnology, Cat # P0018S). Band intensities from Western blots were quantified using ImageJ software (1.54p). For each membrane, target protein signals were normalized to internal reference protein β-actin after background subtraction. Normalized values were then expressed as fold change relative to the control group. All quantifications were performed on blots from at least three independent experiments.

### RNA‑sequencing

Total RNA was extracted from the tissue using TRIzol^®^ Reagent at Shanghai Majorbio Biopharm Technology Co.,Ltd (Shanghai, China) according the manufacturer’s instructions. RNA purification, reverse transcription, library construction and sequencing were performed according to the manufacturer’s instructions (Illumina, San Diego, CA). The RNA seq transcriptome library was prepared following Illumina^®^ Stranded mRNA Prep Ligation from Illumina (San Diego, CA) using 1 µg of total RNA. Shortly, messenger RNA was isolated according to polyA selection method by oligo (dT) beads and then fragmented by fragmentation buffer firstly. Secondly double-stranded cDNA was synthesized using a SuperScript double-stranded cDNA synthesis kit (Invitrogen, CA) with random hexamer primers (Illumina). Then the synthesized cDNA wa subjected to end-repair, phosphorylation and ‘A’ base addition according to Illumina’s library construction protocol. Libraries were size selected for cDNA target fragments of 300 bp on 2% Low Range Ultra Agarose followed by PCR amplified using Phusion DNA polymerase (NEB) for 15 PCR cycles. After quantified by Qubit 4.0, paired-end RNA-seq sequencing library was sequenced with the NovaSeq X plus sequencer (2 × 150 bp read length). Differentially expressed genes (DEGs) between HDM group and lactate treatment group were analyzed using DESeq2. Genes meeting both criteria of an absolute log₂(fold change) ≥ 1.5 and adjusted *P*-value < 0.05 were defined as significantly differentially expressed. The transcriptomics data have been deposited in the Sequence Read Archive database under the accession code: PRJNA148003.

### 16S rRNA sequencing

Six animals were randomly selected from the model group and the lactate-treated group for 16S rRNA sequencing analysis. After the mice were anesthetized and sacrificed, the cecum was isolated by opening the abdominal cavity, and the contents at the distal end of the cecum were taken out, placed into a sterile Eppendorf tube, and immediately stored in a -80 °C cryogenic freezer. Under sterile conditions, 400 mg of fecal samples were weighed and added into 2 mL EP tubes, and genomic DNA was extracted from each sample according to the instructions of the fecal DNA extraction kit. The quality and concentration of the extracted genomic DNA were measured using a Thermo Nanodrop 2000 spectrophotometer and 1% agarose gel. Based on the amplified 16S region characteristics, PCR amplification of the 16S rDNA V3-V4 hypervariable region was performed using primers 338 F (5′-ACTCCTACGGGAGGCAG-3′) and 806R (5′-GGACTACHVGGGTWTCTAAT-3′). Following 2% agarose gel electrophoresis of the amplified products, sequencing was performed on the Illumina NovaSeq platform, yielding 250-bp paired-end sequences. Raw sequencing data were assembled and filtered to obtain clean data. Subsequently, all cleaned data from every sample were clustered using UParse software (UParse v7.0.1001), grouping sequences into operational taxonomic units (OTUs) at 97% sequence similarity.

### Statistical analysis

All experiments were repeated at least 3 times, and data error bars correspond to the standard errors of the mean (SEM). Statistical analysis was performed with GraphPad software (version 8.0; https://www.graphpad.com). The significance of the statistical difference between the 2 groups was analyzed using Student’s t test, while one-way analysis of variance (ANOVA) and 2-way ANOVA were used for analyzing multiple groups.Statistical significance was set at *P* < 0.05.

## Results

### Lactate ameliorated allergic airway inflammation in mice

**Fig. 1:**
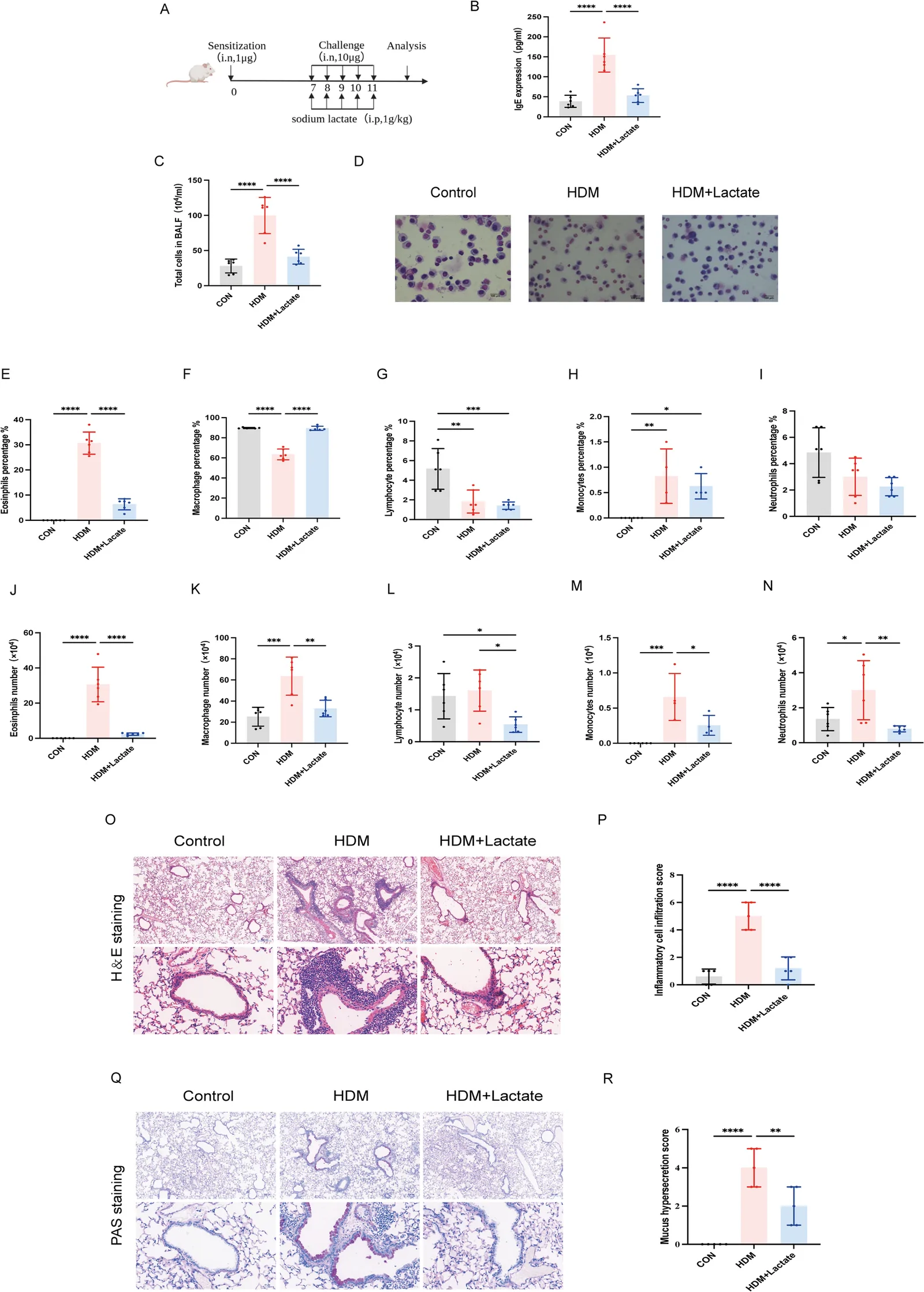
Lactate reduced inflammatory responses in HDM-induced allergic asthma. **A** Establishment of a HDM-induced allergic asthma mouse model and lactate intervention in BALB/c mice. **B** Serum IgE levels. **C** Total cell counts in bronchoalveolar lavage fuid (BALF). **D** Wright’ stainning of BALF smears. **E**-**N** The percentage and absolute number of eosinophils, macrophages, lymphocytes, monocytes, and neutrophils in BALF. **O**-**R** H&E and PAS staining of the indicated lung tissue sections and quantitative analysis of tissue damage. For A-P, *n* = 5–6 per group. Data are representative of three independent experiments. ns: nonsignificant, ^*^p < 0.05, ^**^p < 0.01, ^***^p < 0.001, ^****^p < 0.0001. All data are presented as mean ± SEM

To investigate effect of lactate on allergic asthma, an in vivo protocol for an HDM (the common inhaled allergen that causes allergic asthma)-induced allergic asthma murine model with lactate treatment was established (Fig. 1A). We observed that serum IgE levels were reduced dramatically in the HDM-induced asthma mouse model received lactate (Fig. 1B). In parallel, lactate alleviated total inflammatory cells accumulation in pulmonary (Fig. 1C-D), especially eosinophils, lymphocytes and monocytes numbers in BALF. Although percentage of macrophages was increased in lactate treatment group, absolute macrophage numbers was significantly decreased (Fig. 1E-N). Moreover, histological examinations of lung tissue samples were performed by using hematoxylin and eosin (H&E) and periodic acid-Schiff (PAS) staining. Our data showed that lactate reduced inflammatory cell infiltration, airway wall thickening and mucus production during asthma attack (Fig. 1O-R). Above all, it was implicated that lactate application could relieve allergic airway inflammation and display a protective activities in HDM-induced allergic asthma mice.

### Lactate relieved type 2 responses and EETs formation in pulmonary

**Fig. 2:**
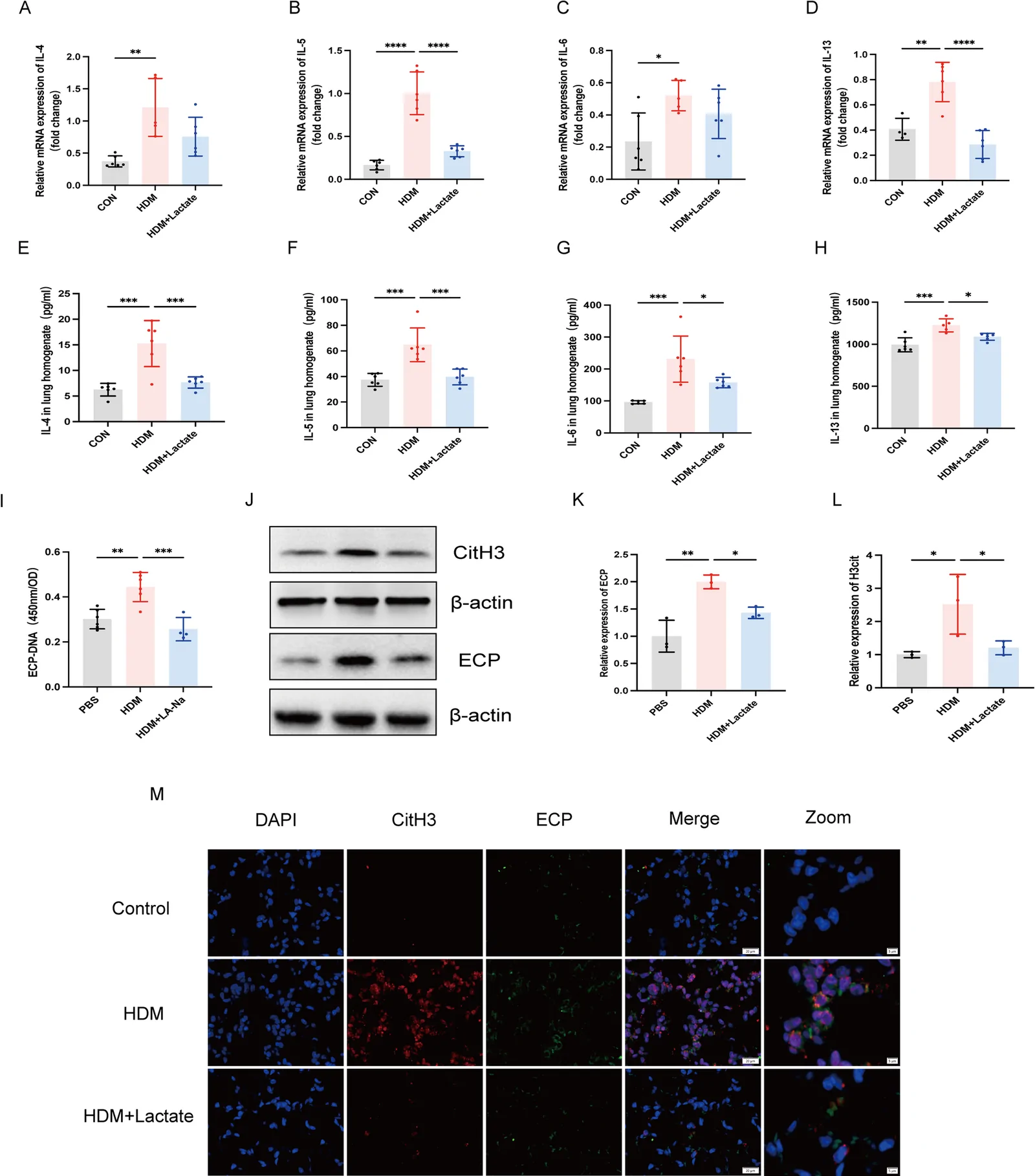
Lactate inhibited type 2 responses and EETs formation. **A**-**D** mRNA levels of IL-4, IL-5, IL-6, and IL-13 in lung homogenates detected by Quantitative real-time PCR. **E**-**H** Levels of inflammatory factors IL-4, IL-5, IL-6, and IL-13 in lung tissues detected by ELISA. **I** ECP-DNA level in BALF measured using ELISA. **J**-L Western blot analysis showing the protein expression of ECP and H3cit. M Representative immunofluorescence images of EETs. Scale bars are included in the images. For (**A**-**M**), *n* = 5–6 per group. Data are representative of three independent experiments. ns: nonsignificant, ^*^p < 0.05, ^**^p < 0.01, ^***^p < 0.001, ^****^p < 0.0001. All data are presented as mean ± SEM

Since type 2 immune responses played an important role in allergic airway inflammation, we aimed to explore the function of lactate in Th2 immune responses. Both mRNA and protein level of Th2 related IL-4, IL-5 and IL-13 as well as pro-inflammatory IL-6 were detected. Consistently, lactate remarkably inhibited IL-4, IL-5, IL-13 and IL-6 in mice lung tissues (Fig. 2A-H). Recently, EETs was reported to be involved in pathogenesis of allergic asthma. We therefore evaluated EETs related marker ECP (eosinophil cationic protein), CitH3 (Citrullinated Histone H3) and ECP-DNA in mice with HDM-induced asthma. Our data indicated that lactate significantly downregulated ECP-DNA levels in lung homogenates (Fig. 2I), together with protein level of ECP and CitH3 in lung tissues (Fig. 2J-L). Moreover, immunostaining was used to analyze EETs formation as evidenced by co-localization of ECP and CitH3 (Fig. 2M), HDM-induced asthmatic mice received lactate treatment exhibited reduced EETs formation.

### RNAseq analysis revealed anti-inflammatory role of lactate

**Fig. 3:**
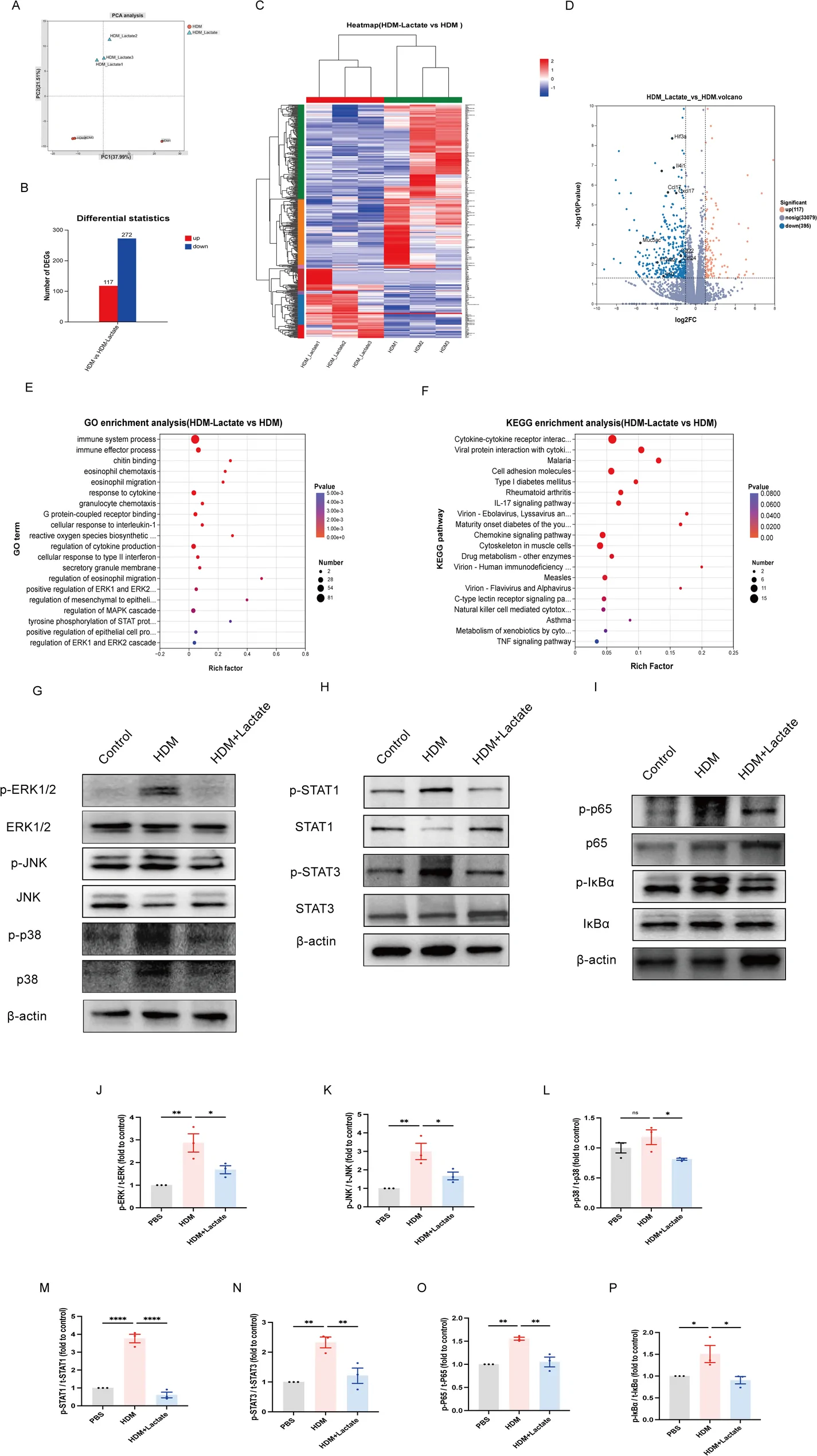
Transcriptomic analysis of differential gene expression between HDM-Lactate and HDM groups. **A** Principal component analysis (PCA). **B** Number of differentially expressed genes (DEGs). **C** Hierarchical clustering heatmap of DEGs. Rows represent genes, and columns represent samples. Red and blue indicate upregulated and downregulated gene expression, respectively. **D **Volcano plot of DEGs. **E** GO enrichment analysis of DEGs. **F** KEGG pathway enrichment analysis of DEGs. **G**-**I** Western blotting analysis of pro-inflammatory related pathways including MAPK, STAT and NF-κB. **J**-**P** Quantative analysis of relative expression phosphorylation of ERK1/2, JNK, p38, STAT1, STAT3, p65 and IκBα at protein levels

To explore the immunoregulatory effect of lactate on allergic asthma, lung tissues of mice were used for RNA sequencing (RNA-Seq) analysis. PCA (Principal component analysis) revealed that lactate treatment resulted in distinct transcriptomic states (Fig. 3A). Moreover, a total of 389 genes were differentially expressed including 117 upregulated genes and 272 downregulated genes (Fig. 3B-C). Volcano plot revealed that lactate treatment significantly decreased type 2 related cytokine and chemokine genes such as IL-4, IL-13, CCL17, CCL22, CCL24 and so on (Fig. 3D). GO enrichment analysis indicated that lactate treatment was closely associated with immune responses and inflammatory cells migration in allergic asthma (Fig. 3E). In addition, KEGG enrichment analysis demonstrated that anti-inflammatory function of lactate was closely associated with MAPK and STAT signaling pathways (Fig. 3F). Furthermore, quantitative analysis of Western blot results (Fig. 3G-P) was applied to validate that lactate significantly ameliorated phosphorylation of MAPK including ERK1/2 (Fig. 3J), JNK (Fig. 3K) and p38 (Fig. 3L), together with STAT1 (Fig. 3M), STAT3 (Fig. 3N), p65 (Fig. 3O) and IκBα (Fig. 3P) pathways when compared to HDM group. These finding suggested that lactate suppressed inflammatory responses via downregulating activation of MAPK, STAT1, STAT3 and NF-κB signal cascades.

### Lactate alleviated airway epithelial barrier dysfunction in asthma mice

**Fig. 4:**
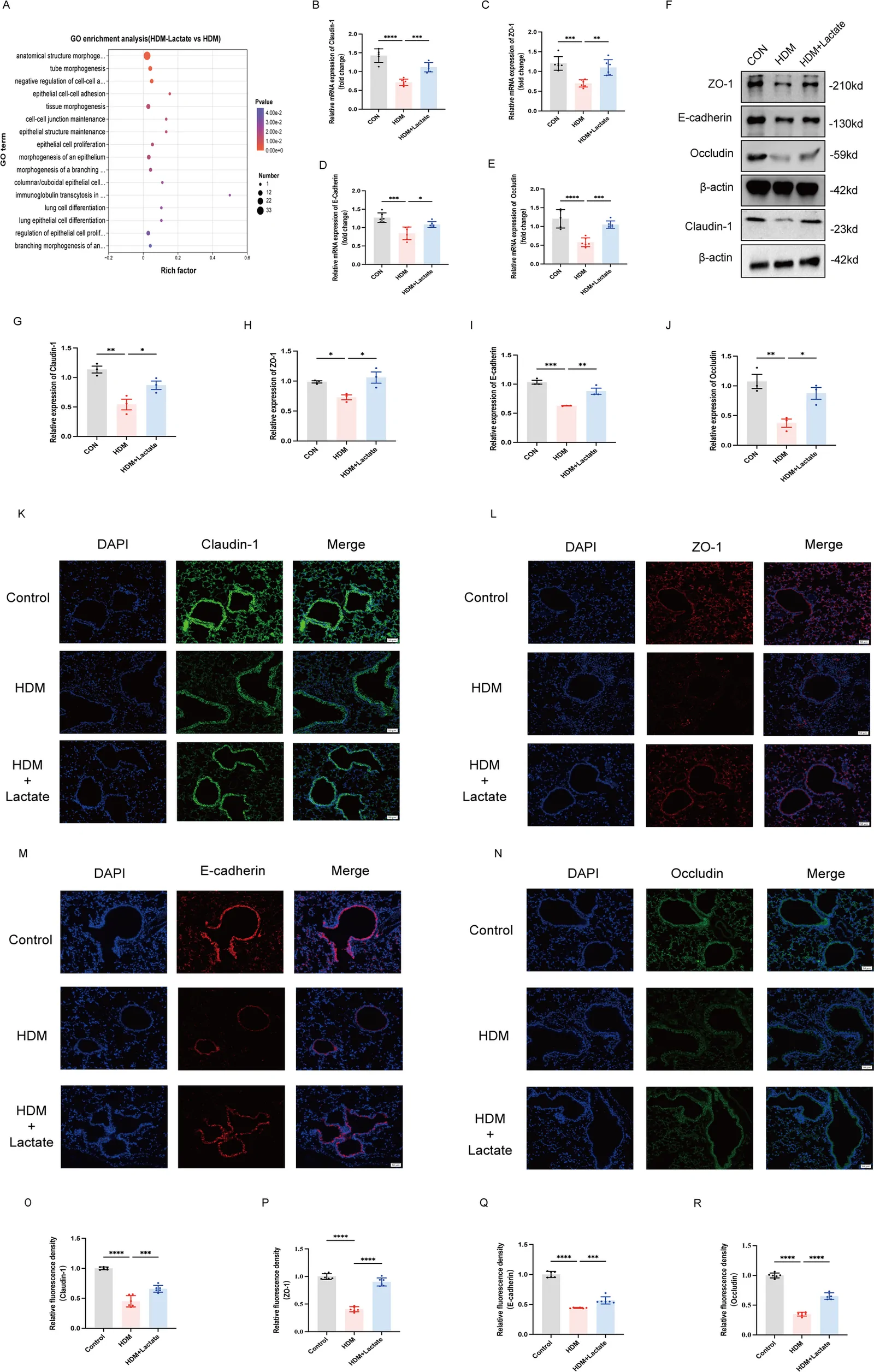
Effects of lactate on epithelial barrier function in the HDM-induced allergic asthma mouse model. **A** GO enrichment analysis of DEGs from the HDM group and Lactate group, showing the top enriched biological processes related to epithelial structure and cell adhesion. **B**-**E** Relative mRNA expression levels of tight junction and adhesion molecules (Claudin-1, ZO-1, E-cadherin, and Occludin) in the control, HDM, and HDM+Lactate groups. **F** Western blot analysis showed the protein expression of ZO-1, E-cadherin, Occludin, and Claudin-1. **G**-**J** Quantative analysis of relative expression of ZO-1, E-cadherin, Occludin, and Claudin-1. Immunofluorescence staining of Claudin-1 (**K**), ZO-1 (**L**), E-cadherin (**M**), and Occludin (**N**) in lung tissue sections. Scale bars are included in the images. **O**-**R** Quantitative analysis of the relative fluorescence density of Claudin-1, ZO-1, E-cadherin, and Occludin. Data are presented as mean ± SEM. Statistical significance: *p < 0.05, ^**^p < 0.01, ^***^p < 0.001, ^****^p < 0.0001

According to GO enrichment analysis, airway epithelial cells played an important role in the HDM-induced asthmatic mice received lactate treatment (Fig. 4A). The respiratory epithelium provides a physical, immunologic and functional barrier to protect host from inhaled environmental particles including allergens, air pollutants and respiratory infections to maintain airway epithelial barrier homeostasis. The expression of key tight and adherens junction genes ZO-1, E-cadherin, occludin and claudin-1 were measured in lung tissues. Our results showed that the mRNA expression level of ZO-1, E-cadherin, occludin and claudin-1 were decreased, while lactate treatment significantly increased adhesion molecules and tight junctions expression in the HDM-induced asthma mouse model (Fig. 4B-E). In addition, protein level of ZO-1, E-cadherin, Occludin and Claudin-1 were confirmed in lactate treatment group by Western blot (Fig. 4F) and immunofluorescent staining (Fig. 4K-N), respectively. Quantitative data of Western blot results indicated that lactate could dramatically upregulate Claudin-1 (Fig. 4G), ZO-1 (Fig. 4H), E-cadherin (Fig. 4I) and Occludin (Fig. 4J) expression. Relative intensity analysis of immunofluorescent staining indicated that lactate was able to upregulate tight junctions and adhesion molecules (Fig. 4O-R). These findings suggested that lactate was able to up regulate adhesion molecules and tight junctions to sustain airway epithelial barrier function.

### Lactate inhibited pulmonary apoptosis in HDM-induced asthma mice through Bcl-2/Bax/ Caspase 3

**Fig. 5:**
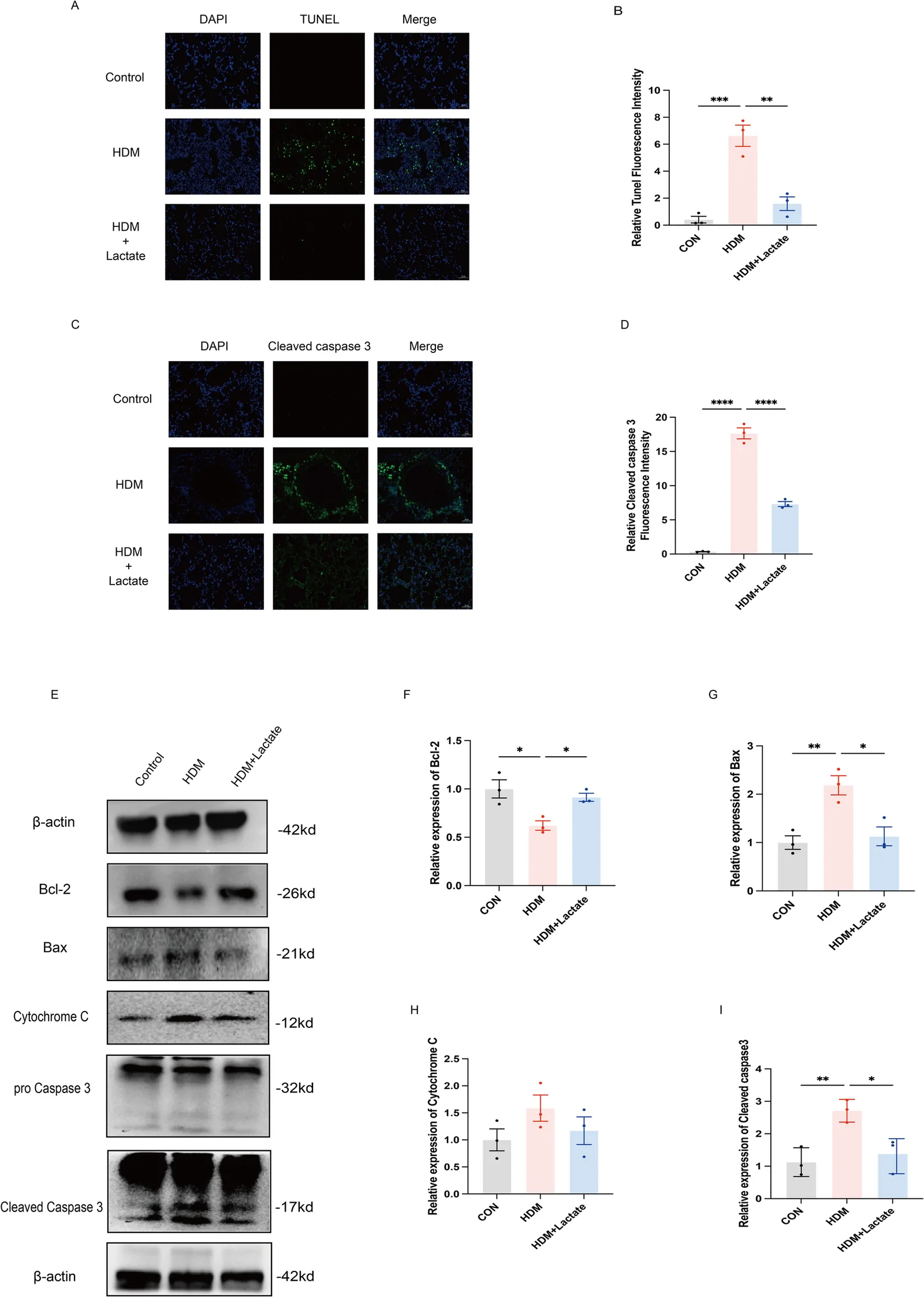
Effects of lactate on HDM-induced airway epithelial cell apoptosis in a mouse model of allergic asthma. **A** Representative images of TUNEL staining in lung tissues from the control, HDM, and HDM+Lactate groups. **B** Quantitative analysis of relative TUNEL fluorescence intensity. **C** Representative immunofluorescence staining of Cleaved Caspase-3 in lung tissues. **D** Quantitative analysis of relative Cleaved Caspase-3 fluorescence intensity. **E** Western blot analysis of apoptosis-related proteins (Bcl-2, Bax, Cytochrome C, and Cleaved Caspase-3) in lung tissues. **F**–**I** Quantification of the relative expression levels of Bcl-2 (**F**), Bax (**G**), Cytochrome C (**H**), and Cleaved Caspase-3 (**I**). All data are presented as mean ± SEM. Statistical significance: ^*^p < 0.05, ^**^p < 0.01, ^***^p < 0.001, ^****^p < 0.0001

It has been reported that apoptosis is closely involved in maintaining airway epithelial barrier function [14]. Therefore, we aimed to investigate whether lactate had an impact on apoptosis. TUNEL assay revealed that lactate application significantly decreased apoptotic cells in lung tissues of HDM-induced asthmatic mice when compared to HDM group (Fig. 5A-B). Consistently, Cleaved Caspase 3 positive cells were reduced by lactate administration as shown by immunofluorescent staining (Fig. 5C-D). Then, we analyzed protein level of apoptosis pathways molecules. The results showed that Bax, cytochrome C and Cleaved Caspase 3 were increased and Bcl-2 were decreased in lung tissues of mice with HDM-induced asthma, while lactate treatment significantly downregulated Bax, cytochrome C and Cleaved Caspase 3 as well as upregulated Bcl-2 (Fig. 5E-I). These results demonstrated that lactate could limit apoptosis through Bcl-2/Bax/ Caspase 3 pathways in asthma mice.

### Lactate modulated gut microbiota in HDM-induced asthma mice

**Fig. 6:**
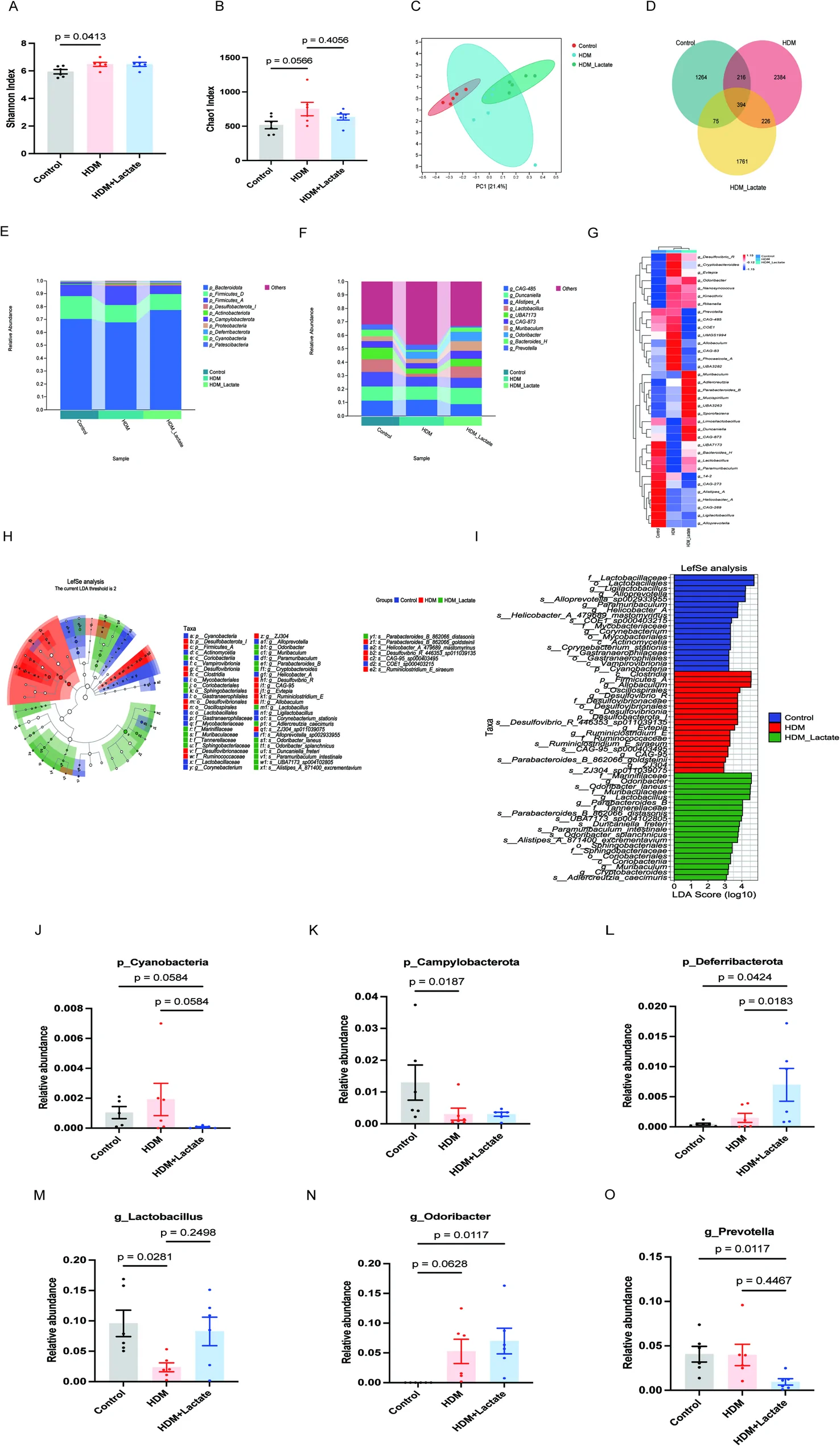
Effects of lactate on the gut microbiota composition in the HDM-induced allergic asthma mouse model. **A** Alpha diversity analysis of gut microbiota, including Shannon index and Chao1 index (**B**), in the Control, HDM, and HDM+Lactate groups. **C** Principal component analysis (PCA) based on Bray-Curtis distance showing the overall structure of gut microbiota across groups. **D** Venn diagram illustrating the overlap of operational taxonomic units (OTUs) among the three groups. **E**, **F** Stacked bar plots showing the relative abundance of gut microbiota at the phylum (**E**) and genus (**F**) levels. **G** Heatmap displaying the relative abundance of the top 30 most abundant genera across the three groups. **H** Circular cladogram from LEfSe analysis showing taxonomic differences in gut microbiota among groups. **I** Linear discriminant analysis (LDA) effect size (LEfSe) identifying key bacterial taxa with significant differences, with an LDA score threshold of 2.0. **J**–**L** Relative abundance of significantly altered bacterial phyla, including Cyanobacteria (**J**), Campylobacterota (**K**), and Deferribacterota (**L**). **M**–**O** Relative abundance of significantly altered bacterial genera, including Lactobacillus (**M**), Odoribacter (**N**), and Prevotella (**O**). Data are presented as mean ± SEM. Statistical significance: ^*^p < 0.05, ^**^p < 0.01, ^***^p < 0.001, ^****^p < 0.0001

Recent studies highlighted that the significance of gut microbiota in allergic asthma, we therefore investigated whether lactate treatment was involved in alterations of gut microbiota through 16S rRNA gene sequencing of fecal samples at the end of study. In terms of alpha diversity, the Shannon index and Chao1 index suggested that the diversity and richness of gut microbiota were different between control and HDM group (Fig. 6A-B). Principal coordinate analysis (PCoA) suggested that unique clustering of microbiota composition for each group (Fig. 6C). To further analyze the relative abundance of gut microbiota, Venn diagram analysis was performed and the relative abundance of gut microbiota at various taxonomic levels was assessed (Fig. 6D). As shown in Fig. 6E-F, the top 10 microbial communities at the phylum and genus levels was displayed, indicating notable alterations in gut microbiota following HDM sensitization and challenge. It was worth noting that microbial profile of lactate group was similar to control group. Heatmap analysis of the top 35 genera showed increased abundance of *Lactobacillus*, *Limosilactobacillus* and *Bacteroides*, showing a shift towards a healthier state (Fig. 6G). At the phylum level, LEfSe analysis identified that a marked predominance of *p_Firmicutes_A* and *p_Desulfobacterota_I* in HDM group (Fig. 6H). At the genus level, the LDA score demonstrated that *g_Ligilactobacillus* and *g_Alloprevorella* were enriched in control group, *g_Allobaculum* was enriched in HDM group, together with *g_lactobacillus*, *g_odoribacter* and *g_Parabacteroides_B* were enriched in lactate group (Fig. 6I). In addition, *p_Campylobacterota* was significantly decreased in HDM group when compared to control group, while it was slightly increased in lactate group at phylum levels (Fig. 6K). There was an increase of *p_Cyanobacteria* in HDM group, which was reduced in lactate treatment (Fig. 6J). At genus level, *g_Lactobacillus* was markedly decreased in HDM group compared with control group (Fig. 6M). Moreover, lactate treatment increased abundance of *g_Lactobacillus* (Fig. 6M) and *g_odoribacter* (Fig. 6N) and decreased abundance of *g_Prevotella* (Fig. 6O).

## Discussion

Allergic asthma is a chronic inflammatory disease with high heterogeneity in clinical manifestation and response to treatment, thus there are still huge challenges for asthma control and management [15]. Over the past decades, the treatment of allergic asthma has shifted from bronchodilators and inhaled corticosteroids to more precise approaches targeting specific mechanism in the pathophysiology of asthma. Despite these advances, many patients continue to suffer from asthma attacks and even fail to achieve any degree of remission [16]. Therefore, it is an urgent need to develop effective therapeutic strategies for allergic asthma.

Nowadays, increasing evidence indicates that dysregulated metabolism is involved in the pathophysiology of allergic asthma. It is worth noting that recent advances in glucose metabolism have highlighted the links between glucose homeostasis and disease development. It has been reported that enhanced glycolysis accelerates airway inflammation and airway remodeling as increased oxidative conditions in lung [13]. Lactate, a critical product of glycolysis, was found significantly upregulated in adult and pediatric asthma patients, as well as both HDM and OVA induced asthma mouse models [17]. However, studies on its underlying function in allergic asthma are still insufficient. Allergic asthma is characterized by Th2 inflammatory responses with infiltration of eosinophils, B cells and macrophages in pulmonary together with increased release of type 2 cytokines and IgE [18]. Therefore, we first measured serum level of immunoglobulins in mice with HDM-induced asthma, we found that lactate could dramatically decrease IgE concentration. Additionally, reduced infiltrated inflammatory cells such as eosinophils, macrophages and lymphocytes were observed in lactate treatment group. Histopathological analysis was further confirmed anti-inflammatory role of lactate in allergic asthma. Next, Th2 related cytokines including IL-4, IL-5 and IL-13 were detected in lung tissues, lactate treatment was able to alleviate Th2 responses in HDM-induced mice. Recent studies revealed that eosinophils were reported to form extracellular traps to interact with other immune cells to exacerbate inflammatory responses [19]. Eosinophil extracellular traps consist of eosinophil-derived granule proteins including ECP, eosinophil derived neurotoxin (EDN), eosinophil peroxidase (EPO) and major basic protein (MBP) together with DNA fibers and CitH3 [20]. Moreover, EETs has been regarded as a potential biomarker in allergic diseases [21]. In our current study, lactate could significantly inhibit EETs related ECP-DNA concentrations in mouse lung homogenates. In accordance with previously published studies [19–21], we employed eosinophil cationic protein (ECP) and citrullinated histone H3 (CitH3) as established markers to identify EETs. Reduced ECP-DNA complexes, decreased ECP and CitH3 protein expression, and diminished co-localization of ECP and CitH3 detected by immunofluorescence staining in the lactate treatment group, which indicated that lactate was able to inhibit EETs formation in lung tissues. To further substantiate our findings on EETs quantification, future studies will incorporate ultrastructural visualization and quantitative analysis of extracellular DNA networks. Above all, these data suggested that lactate treatment exerted an immunoregulatory role in allergic airway inflammation. After literature review, we found that lactate played protective effects in several inflammatory conditions. For instance, lactate viewed as a biomarker of severity in critical illness, has recently emerged as a therapeutic target. Moreover, lactate reduces inflammation and protected against cardiac dysfunction and capillary leakage in mouse model of sepsis [22, 23]. Similarly, exogenous lactate attenuates pancreatitis via inhibition of systemic inflammatory responses [24]. However, precise mechanisms involving lactate in inflammatory settings are needed to be illustrated.

Then, RNAseq analysis was applied to elucidate the underlying mechanism of lactate in allergic asthma. According to analyze differentially expressed genes, type 2 cytokines IL-4 and IL-5, eotaxin and chemokines were downregulated in the HDM-induced asthmatic mice treated with lactate. Consistently, Go and KEGG enrichment analysis indicated that lactate was involved in regulation of inflammatory mediators release and immune responses. Next, classical inflammatory signaling cascades including ERK1/2, JNK, p38, NF-κB, STAT1 and STAT3 pathways were verified by western blot, indicating that lactate primarily participated in downregulating inflammatory signaling pathways to decrease proinflammatory cytokines and chemokines production, therefore suppressing allergic airway inflammation. These inflammation related signal cascades were also reported in other mouse disease models with lactate treatment. In a mouse model of hepatic ischemia-reperfusion injury (HIRI), lactate administration inhibited inflammation, migration, oxidative stress, and NETs formation. Mechanistically, lactate decreased Akt/c-Fos and JAK-STAT3 to inhibit IL-33/ST2 pathways, thereby alleviating neutrophil-driven inflammation [25]. In a mouse model of liver and pancreatic injury, lactate prevented activation of NF-κB to reduce inflammation and organ injury [6]. In addition to inflammatory pathways, lactate could mitigate multiorgan dysfunction and suppresses inflammatory infiltrates in sepsis by histone lactylation [26]. In all, anti-inflammatory action of in lactate in allergic airway inflammation was associated with downregulation of proinflammatory signal cascades activation.

Notably, GO enrichment analysis indicated that lactate was involved in maintenance of epithelial structure and morphogenesis to protect airway epithelial barrier homeostasis. Then, we measured expression level of Claudin-1, ZO-1, E-cadherin and Occludin at both mRNA and protein level. The results showed that lactate significantly increase adhesion molecules and tight junctions expression to ameliorate airway epithelial barrier dysfunction. Airway epithelial cell apoptosis is a critical contributor to the disruption of the bronchial epithelial barrier. Studies on allergic airway inflammation mechanisms have revealed that neutrophil extracellular traps (NETs) could disrupt airway epithelial barrier function partially by inducing airway cell apoptosis, leading to decreased E-cadherin and tight junction genes claudin- 8 and 11 [27]. Additionally, the pleiotropic Th2 cytokine IL-13 increased mitochondrial ROS production and subsequent apoptosis in respiratory epithelial cells. This apoptosis was directly associated with loss of epithelial barrier function and mitochondrial calcium uniporter (MCU) inhibition prevented both apoptosis and barrier dysfunction, as well as preserved tight junction protein ZO-1 expression in vivo [28]. In our current study, increased EETs and IL-13 were observed in lung tissues of HDM-induced asthmatic mice, which might contribute to epithelial cell apoptosis. In addition, MAPK, STAT, and NF-κB signaling pathways intricately regulate the mitochondrial apoptotic pathways by controlling the expression and activity of key effector molecules including Bax, Bcl-2, cytochrome C, and Caspase-3. Specifically, the JNK and p38 MAPK cascades promoted apoptosis by directly phosphorylating and activating the pro-apoptotic protein Bax, inducing its translocation to the mitochondrial membrane, while simultaneously suppressing anti-apoptotic Bcl-2 expression [29]. STAT1 acts as a pro-apoptotic transcription factor that directly binds to the Bax promoter to increase its expression [30]. NF-κB signaling pathways was widely reported to participate in apoptotic process. For instance, galectin-3 derived from macrophages contributed to apoptosis through TLR4/MyD88/NF-κB/NLRP3 during atherosclerosis [31]. Although MAPK, STAT, and NF-κB signaling are often studied in the context of inflammation and apoptosis, their sustained activation is closely associated with epithelial barrier disruption. JNK and p38 MAPK signaling promote epithelial injury by downregulating tight junction proteins and adhesion molecules, including ZO-1, Occludin, Claudin-1, and E-cadherin, while NF-κB activation amplifies inflammatory injury and compromises epithelial integrity [32]. Similarly, STAT1 and STAT3 signaling have been implicated in epithelial dysfunction and barrier permeability in several inflammatory diseases [33, 34]. Therefore, inhibition of these pro-inflammatory pathways by lactate likely contributes to the preservation of epithelial junctional complexes, attenuation of epithelial apoptosis, and restoration of airway barrier integrity. Taken together, exogenous lactate exerted a protective role in allergic airway inflammation mediated by suppressing MAPK, STAT, and NF-κB pathways activation, leading to reduced type 2 inflammation and improved airway barrier function.

Recently, gut microbiota has become a “hot spot” in airway allergic inflammation as metabolites from gut can regulate physiological activities and immune status in pulmonary through “gut-lung” axis [35]. There was a divergence in gut microbiota between asthmatic and nonasthmatic subjects. Of recent interest are studies showing that asthmatic patients with eosinophilic inflammatory phenotype demonstrate enrichment in *Fusobacterium* and *Porphyromonas* and decreased relative abundance of *Lactobacillales* order [36]. Similarly, we found that g_*Lactobacillus* was significantly reduced in HDM-induced asthma mice, and lactate supplement could slightly upregulate its abundance. *Lactobacillus* Species was regarded as probiotics in allergic diseases, administration of lactic acid producing bacterial displayed immunomodulatory action and attenuated airway allergic inflammation. In addition, lactate treatment could significantly decrease *Oscillospiraceae* abundance in HDM group. As the increase in the relative abundance of *Oscillatoria* meant the decline of the immune function of the children, suggesting lactate was able to enhance immune responses in asthma mice [37]. Moreover, we observed that dramatically increased *Desulfovibrionia* in HDM group compared to control group. Studies reported that overgrowth of sulfide-producing bacteria was linked to disrupt the integrity of epithelium barrier, triggering inflammation [38]. HDM-induced asthmatic mice receiving lactate treatment displayed lower enrichment of *Desulfovibrionia.* These findings indicated that lactate might improve the composition of the intestinal flora to exert anti-inflammatory activity via gut-lung axis. Since microbiota analysis was performed at the endpoint of the experiment, the current data demonstrate association rather than causation. Several lines of evidence suggest that lactate may influence the gut microbiota indirectly through modulation of host immune responses. Lactate is a well-recognized signaling metabolite that exerts immunomodulatory effects through receptors such as GPR81 and regulates inflammatory pathways including NF-κB, STAT, and MAPK signaling [39, 40]. Therefore, the observed microbial alterations may arise secondary to improvements in systemic immune homeostasis and reduced inflammatory burden. Conversely, it is also plausible that the restoration of beneficial microbial communities contributes to the anti-inflammatory effects of lactate through the gut-lung axis, thereby further attenuating allergic airway inflammation. Future experiments, including fecal microbiota transplantation, antibiotic-mediated microbiota depletion, germ-free animal models, together with longitudinal microbiome analyses will be employed to elucidate the mechanism involving effect of lactate on gut microbiota.

It is remarkable that increased endogenous lactate is reported in asthmatic patients, which may reflect enhanced glycolytic reprogramming, tissue hypoxia, or inflammatory metabolic stress and therefore serve as a disease-associated biomarker. In contrast, exogenous lactate administered under controlled experimental conditions may activate anti-inflammatory or barrier-protective pathways, potentially through lactate-sensitive signaling mechanisms such as GPR81-dependent immunoregulation. Thus, elevated lactate in disease does not necessarily indicate a pathogenic role; rather, lactate may represent both a metabolic marker of inflammation and a regulatory metabolite whose biological effect depends on concentration, timing, cellular target, tissue compartment, and route of delivery. Thus, there are still some limitations in our study. First, the therapeutic window of lactate on airway allergic inflammation remains to be established, further experiments are required to validate its efficacy in reducing type 2 inflammation under therapeutic conditions. Second, the route of lactate delivery needs to be optimized to determine the most efficacious and safe route (local or systemic administration) for therapeutic intervention in allergic airway inflammation. Finally, a positive control such as dexamethasone should be included in future studies to evaluate the efficacy of lactate against current clinical therapies.

In conclusion, our findings elucidated protective effect of lactate in allergic airway inflammation. We observed that decreased inflammatory cell infiltration and improved asthma symptoms in a mouse model of asthma with lactate treatment. In addition, lactate was able to alleviate EETs formation and Th2 related cytokines via inhibiting MAPK, STAT1, STAT3 and NF-κB activation to ameliorate allergic airway inflammation. Moreover, lactate was involved in maintaining airway epithelial cell barrier function and reducing pulmonary apoptosis in the HDM-induced asthmatic mice. Then, gut mirobiota analysis revealed that microbial profile of asthma mice treated by lactate was similar to control group when compared to HDM group. In all, this study demonstrated the regulatory role of lactate in allergic airway inflammation and provides a new perspective for developing therapeutic modality for the treatment of allergic diseases.

## Supplementary Information

Supplementary Material 1.

## Data Availability

No datasets were generated or analysed during the current study.
