# Supplementary material for: Protective role of lactate in allergic airway inflammation: mitigation of inflammatory injury, epithelial barrier integrity restoration, and gut-lung axis involvement
Source: Respir Res. 2026 Jul 13;27:387. doi: 10.1186/s12931-026-03806-z (PMC13644108; doi:10.1186/s12931-026-03806-z)
Supplement: Supplementary file 1 [file 12931_2026_3806_MOESM1_ESM.pdf]

Figure 2J

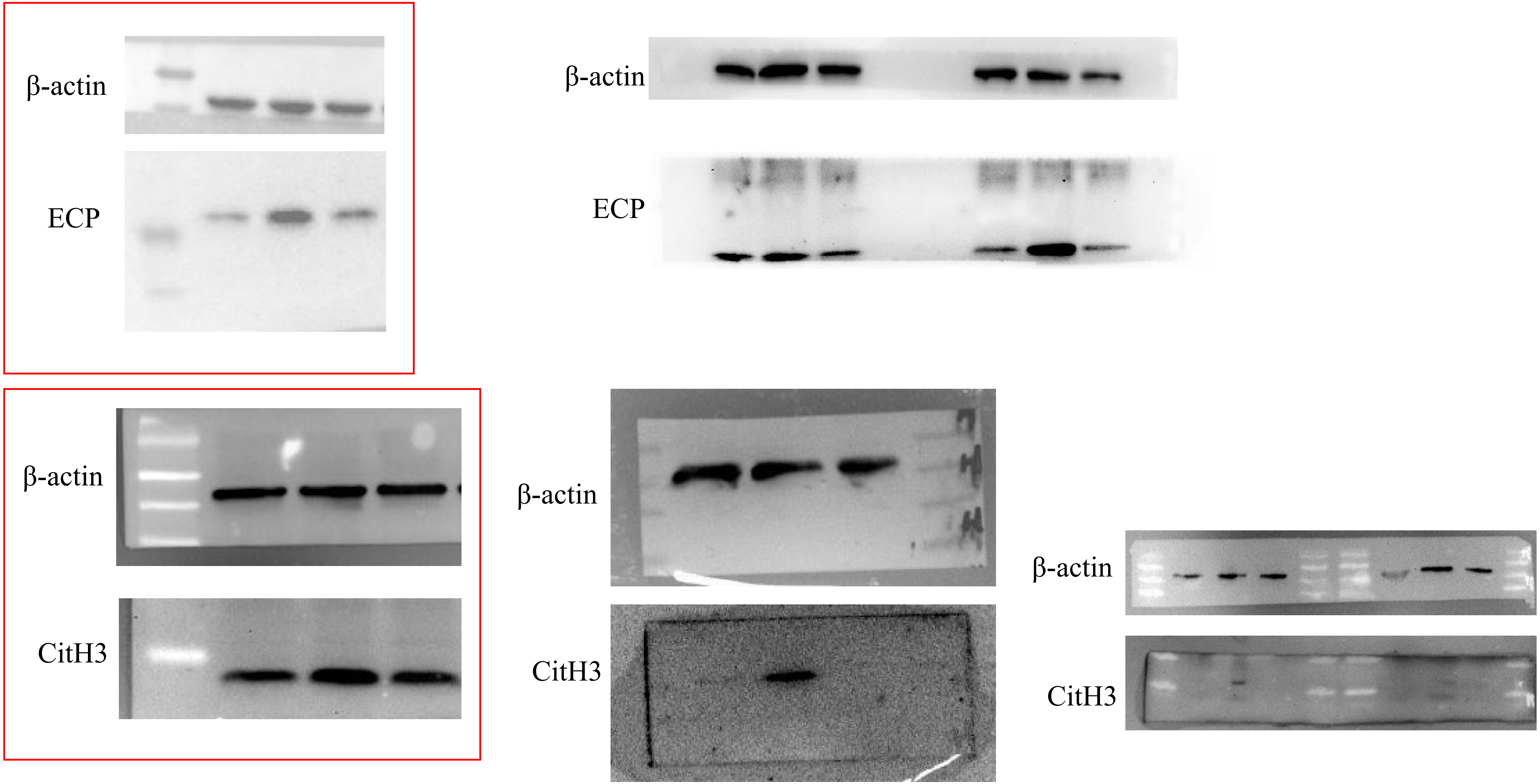
β-actin
ECP
β-actin
ECP
β-actin
CitH3
β-actin
CitH3
β-actin
CitH3

Figure 3G-H

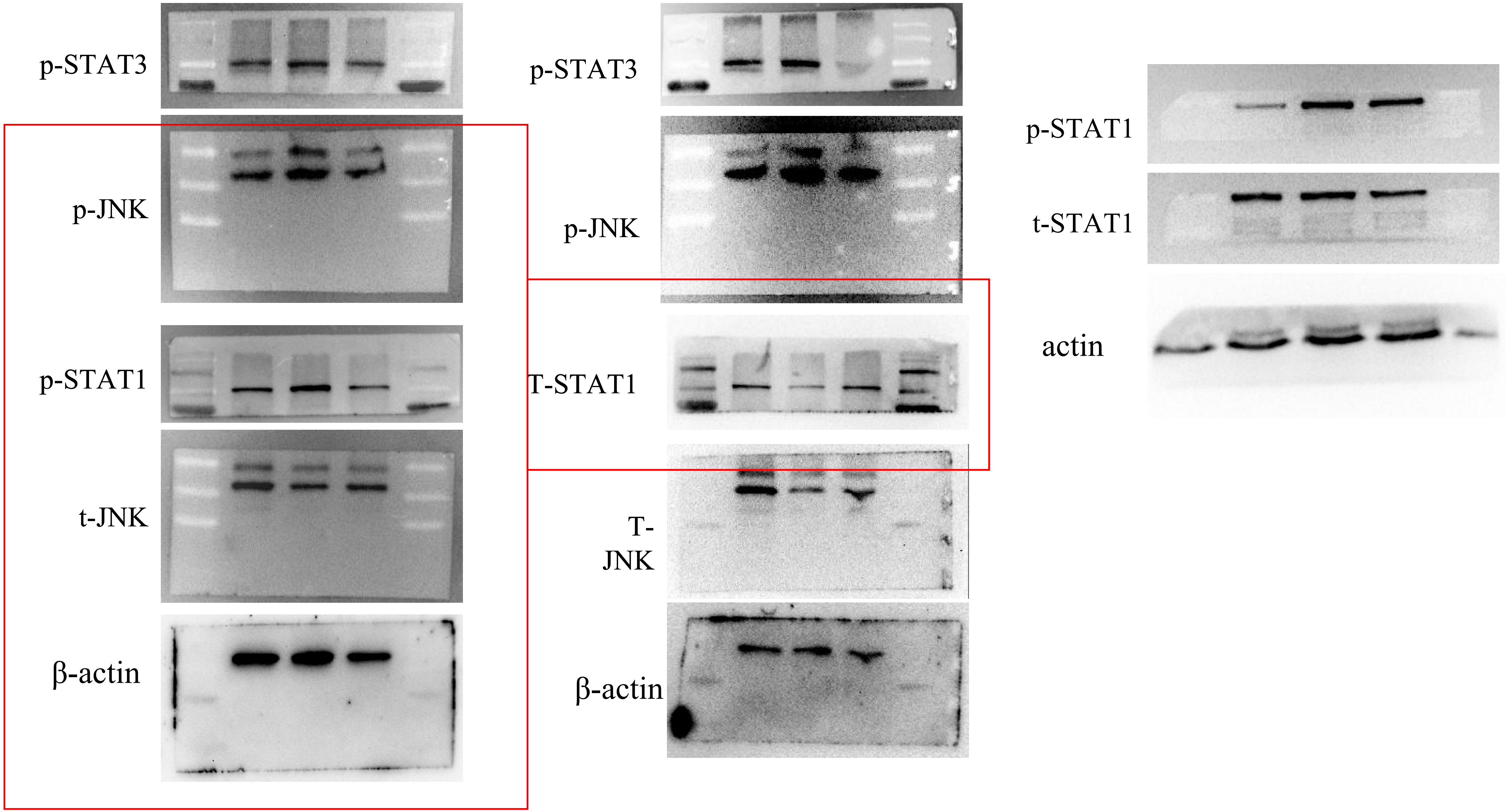
p-STAT3
p-JNK
p-STAT1
t-JNK
β-actin
p-STAT3
p-JNK
T-STAT1
T-
JNK
β-actin
p-STAT1
t-STAT1
actin

Figure 3G

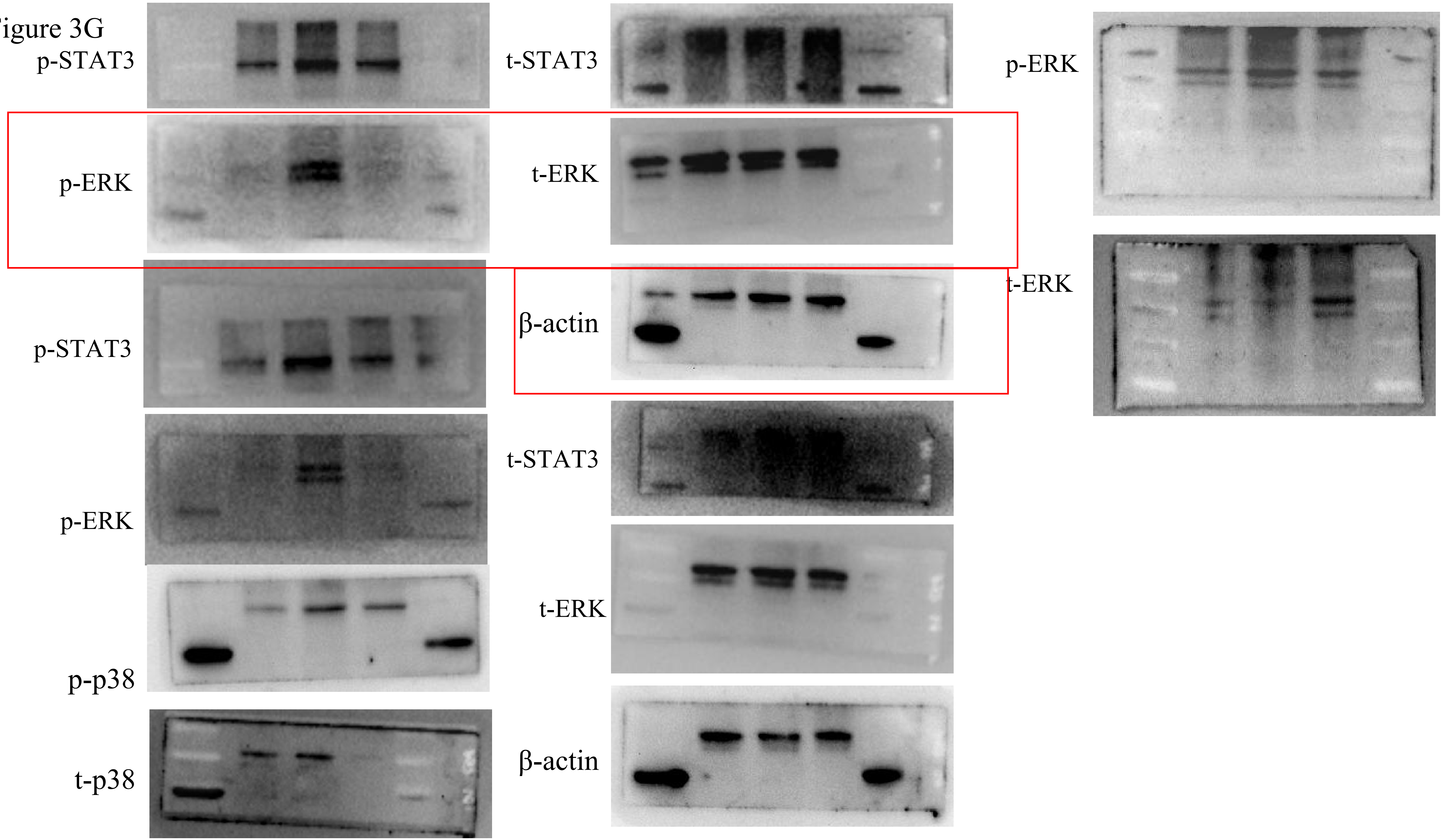
p-STAT3
t-STAT3
p-ERK
p-ERK
t-ERK
t-ERK
p-STAT3
β-actin
t-STAT3
p-ERK
t-ERK
p-p38
t-p38
β-actin

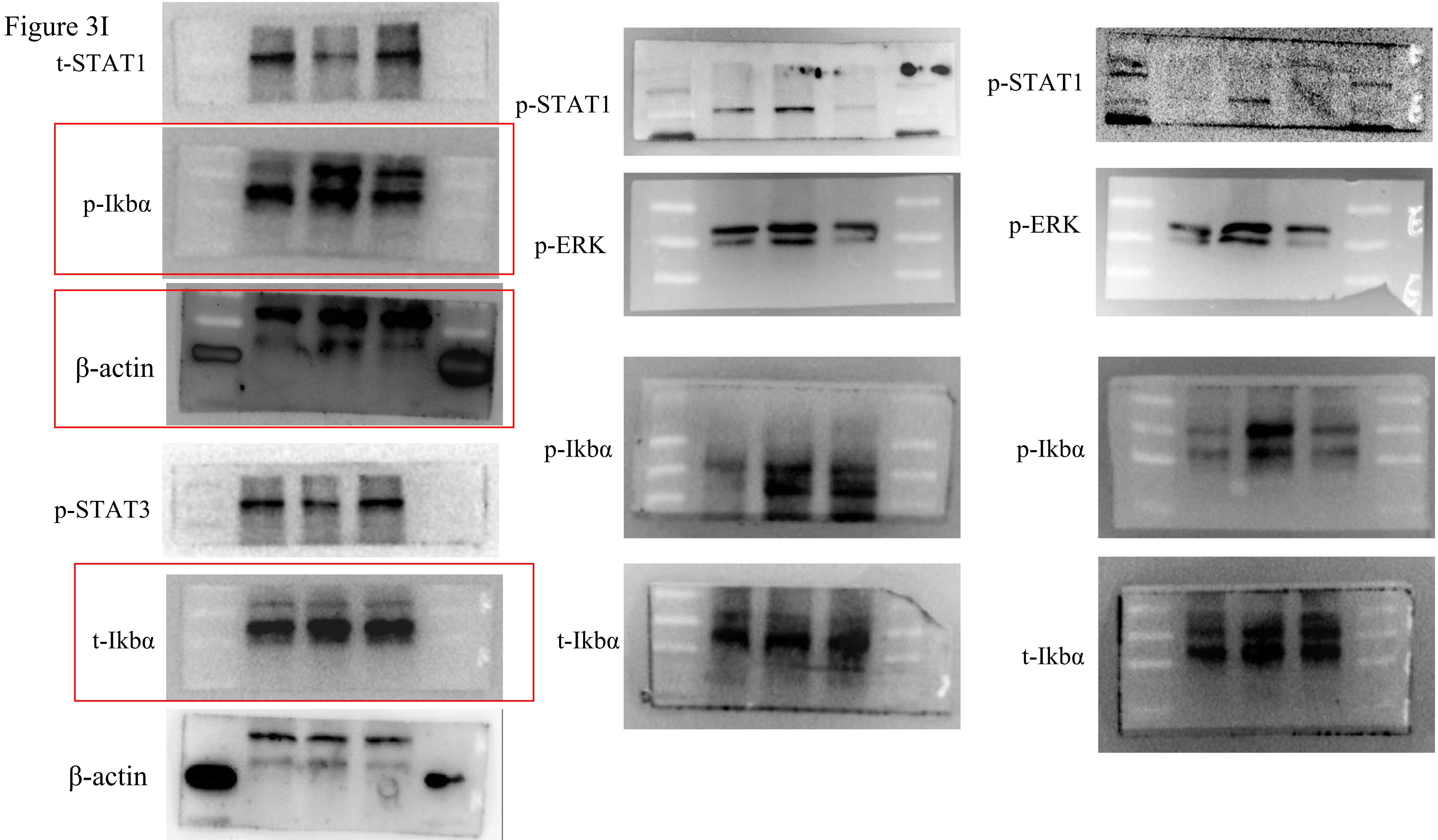
Figure 3I
t-STAT1
p-Ikbα
β-actin
p-STAT3
t-Ikbα
β-actin
p-STAT1
p-ERK
p-Ikbα
t-Ikbα
p-STAT1
p-ERK
p-Ikbα
t-Ikbα

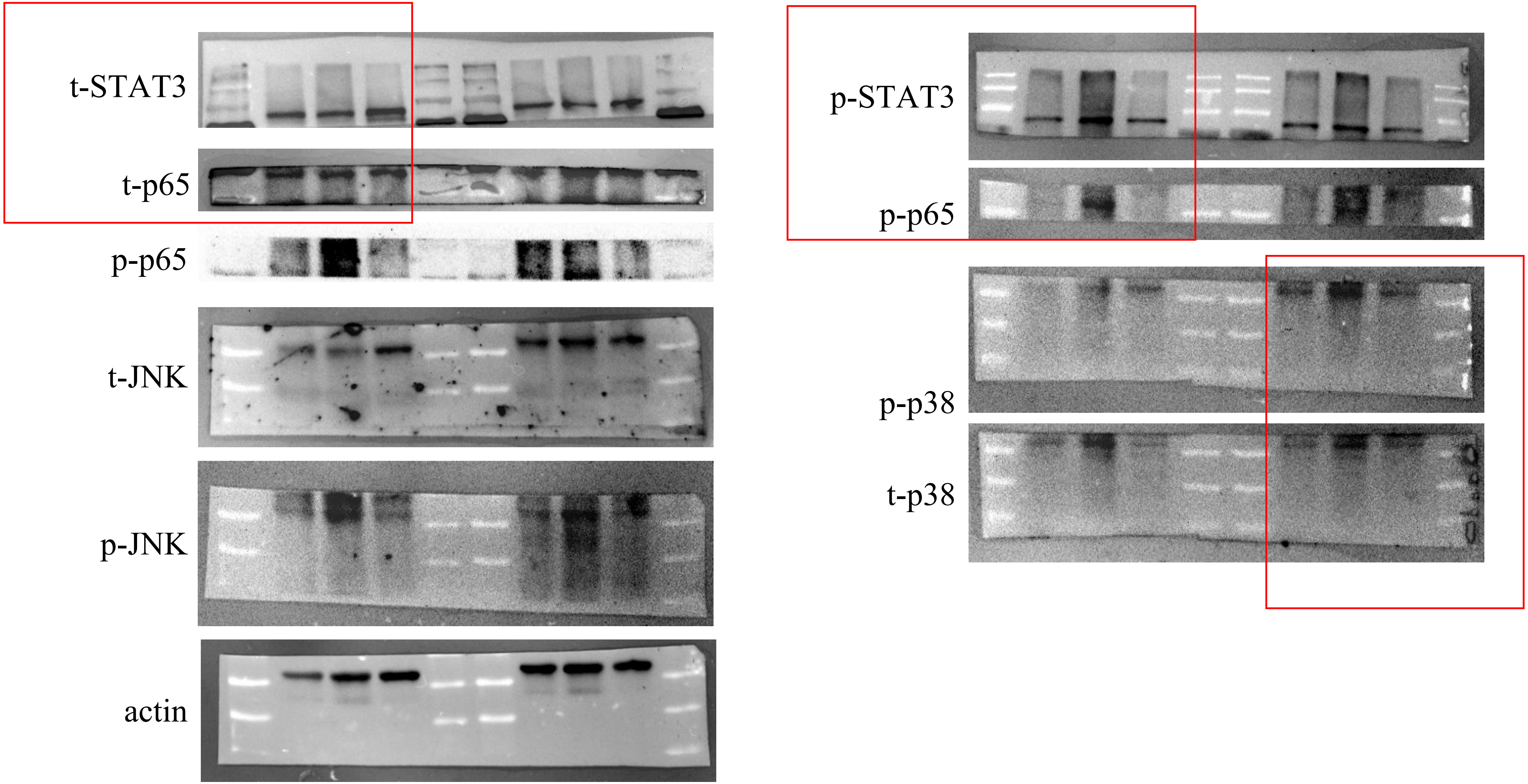

t-STAT3
t-p65
p-p65
t-JNK
p-JNK
actin
p-STAT3
p-p65
p-p38
t-p38

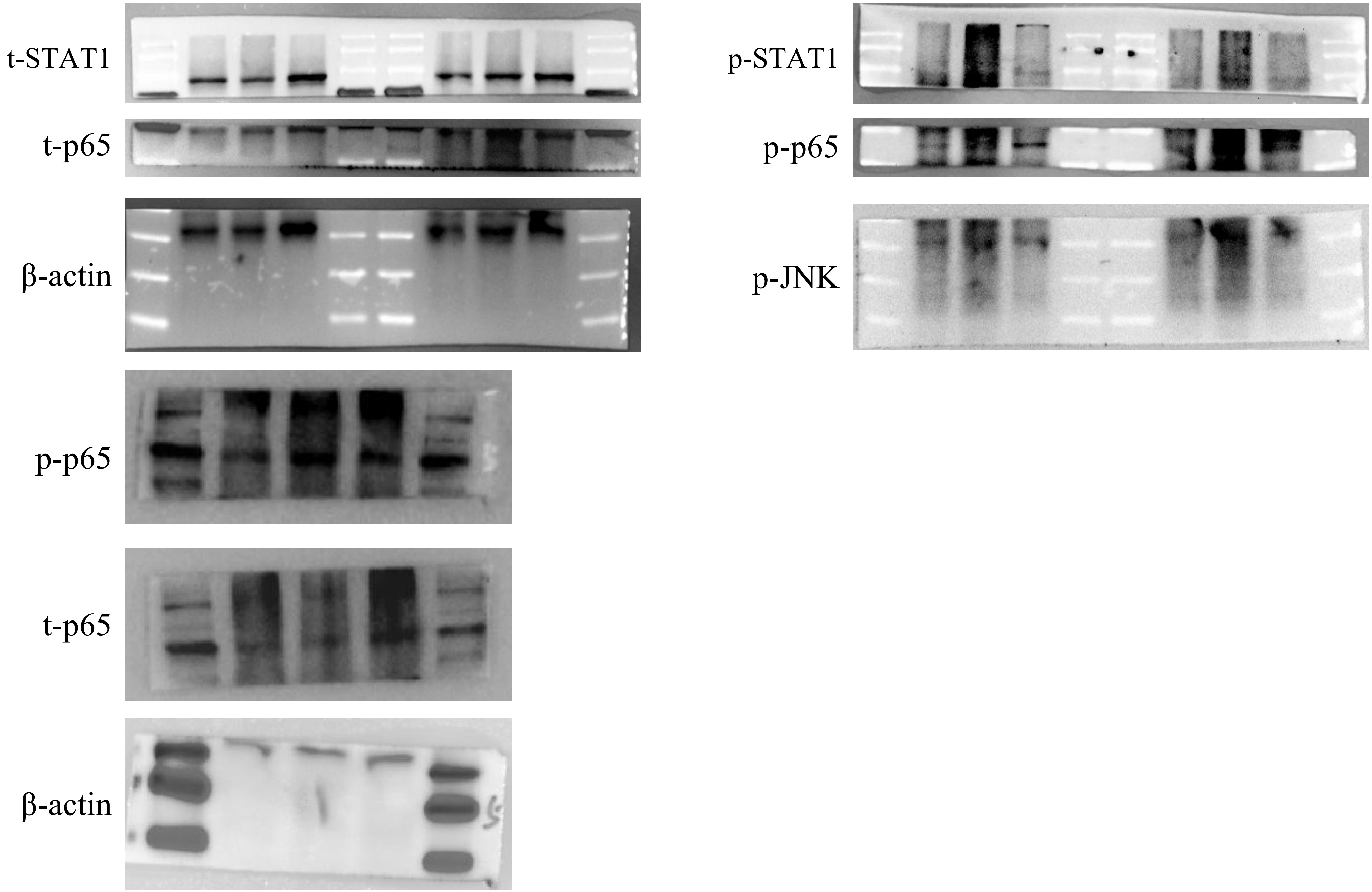
t-STAT1
t-p65
β-actin
p-p65
t-p65
β-actin
p-STAT1
p-p65
p-JNK

Figure 4F

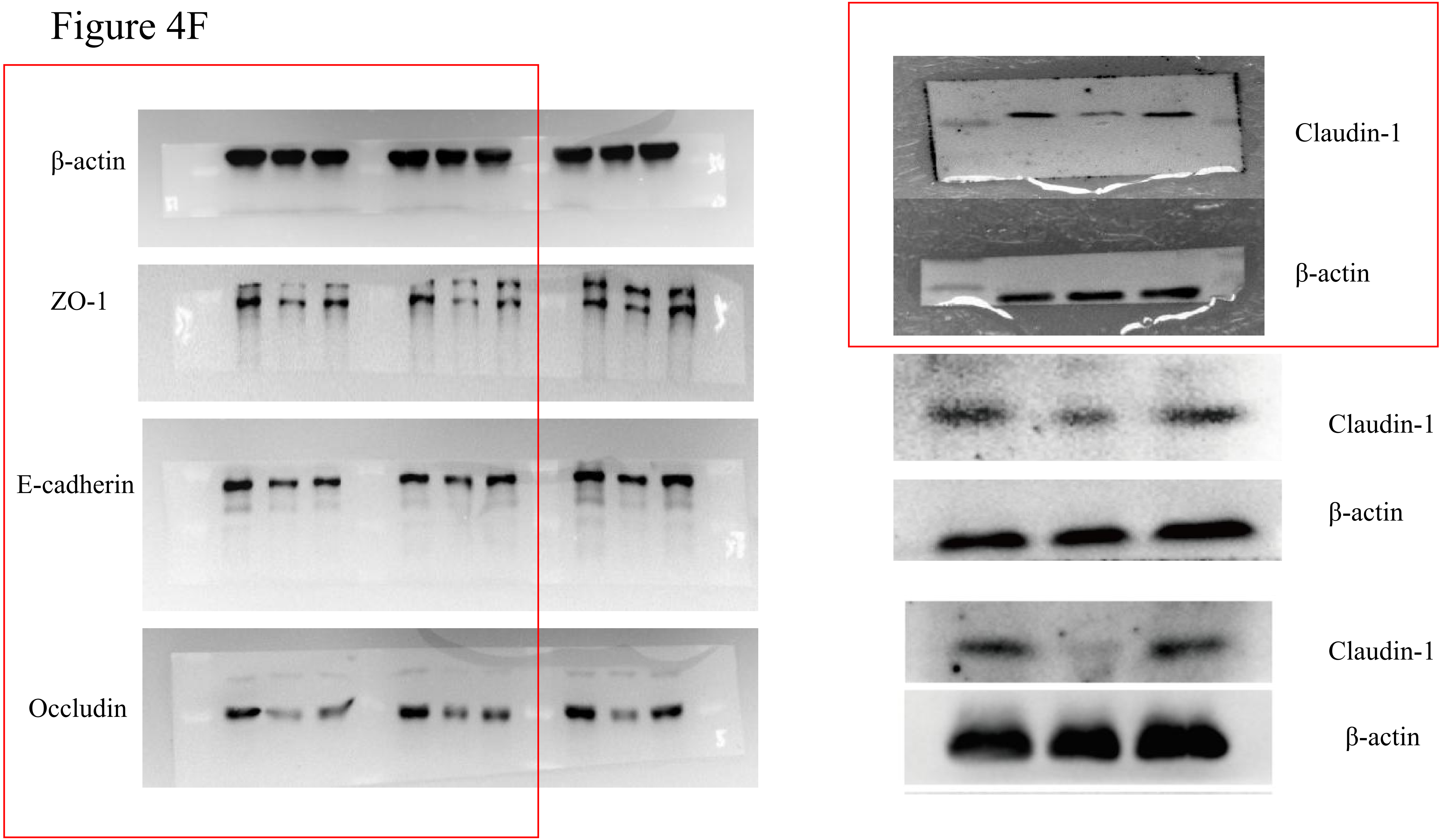
β-actin
ZO-1
E-cadherin
Occludin
Claudin-1
β-actin
Claudin-1
β-actin
Claudin-1
β-actin

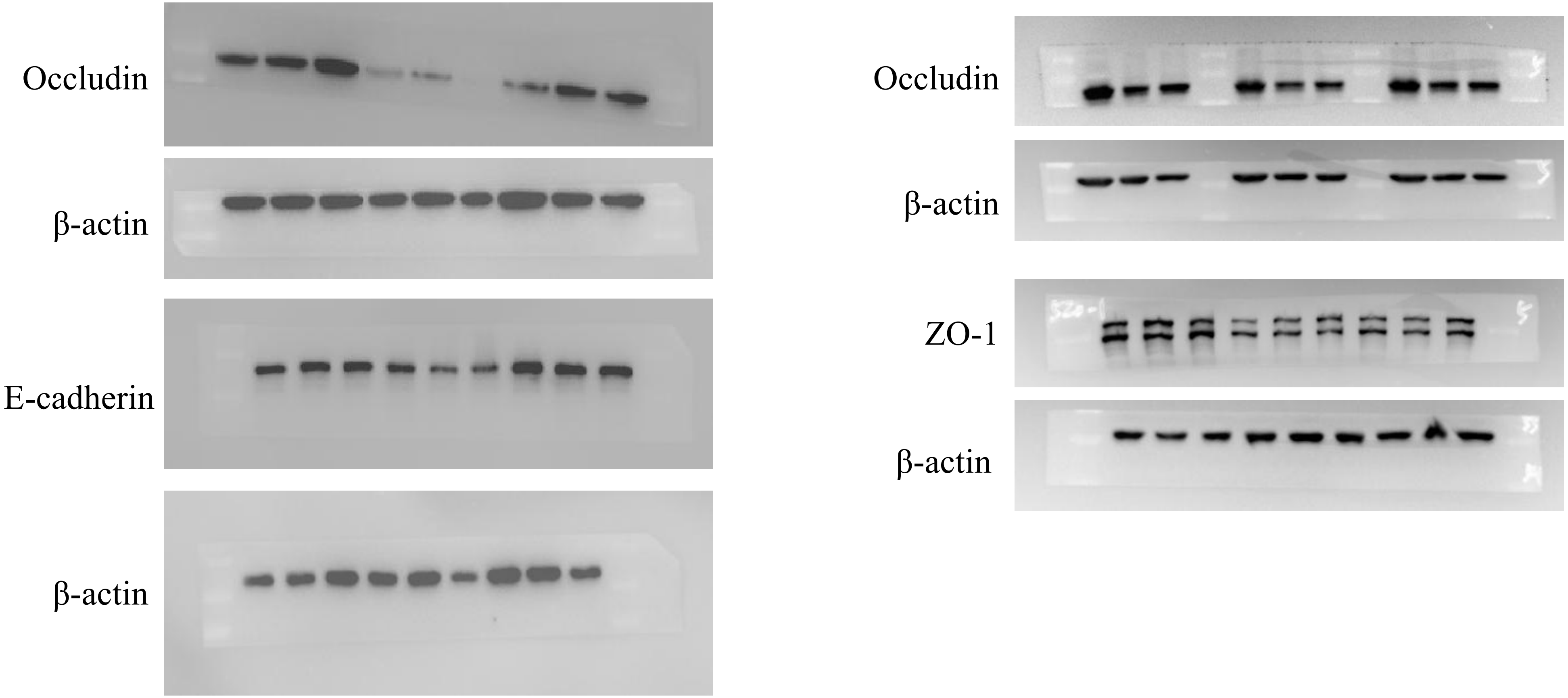
Occludin
β-actin
E-cadherin
β-actin
Occludin
β-actin
ZO-1
β-actin

Figure 5E

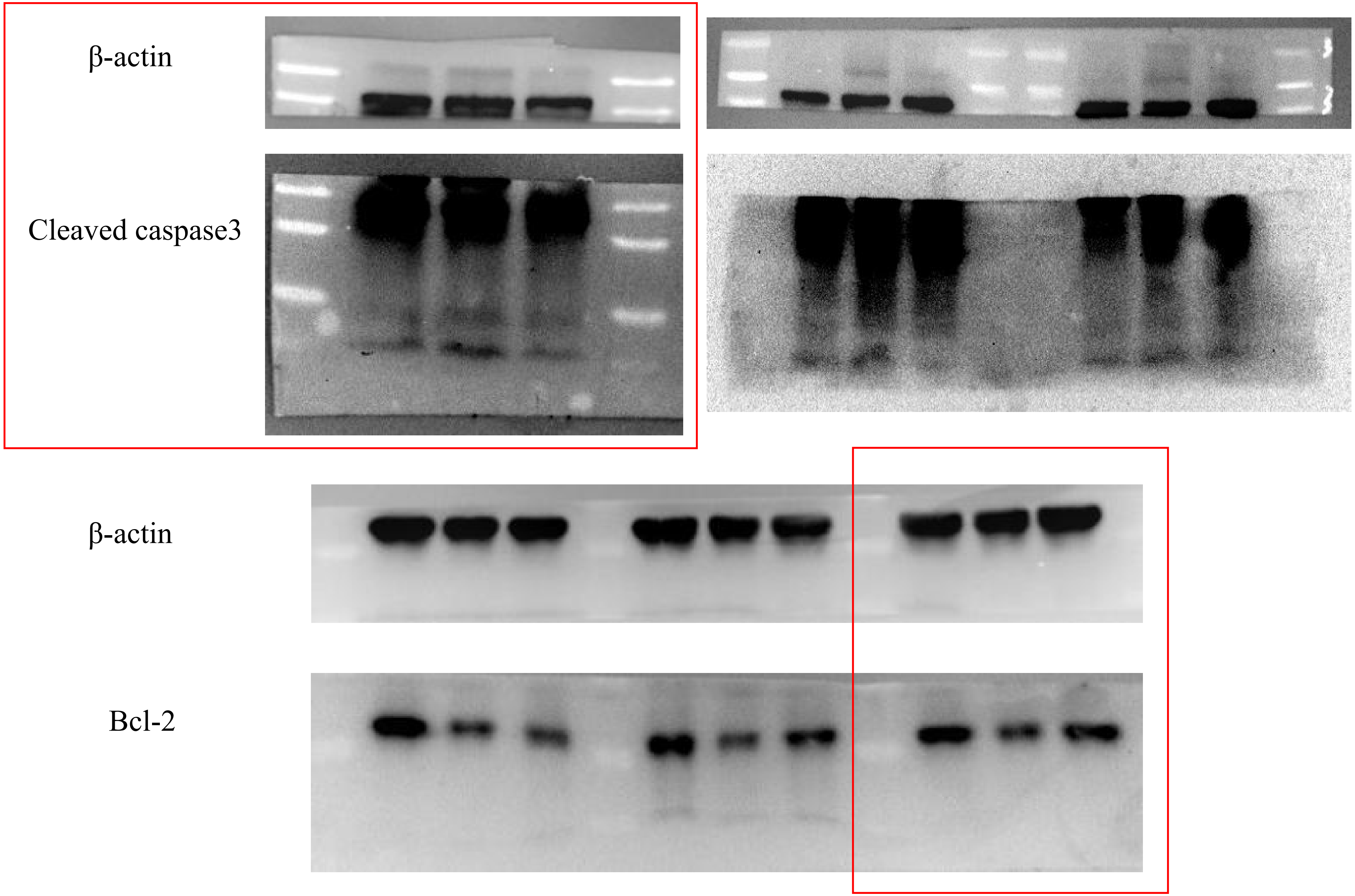

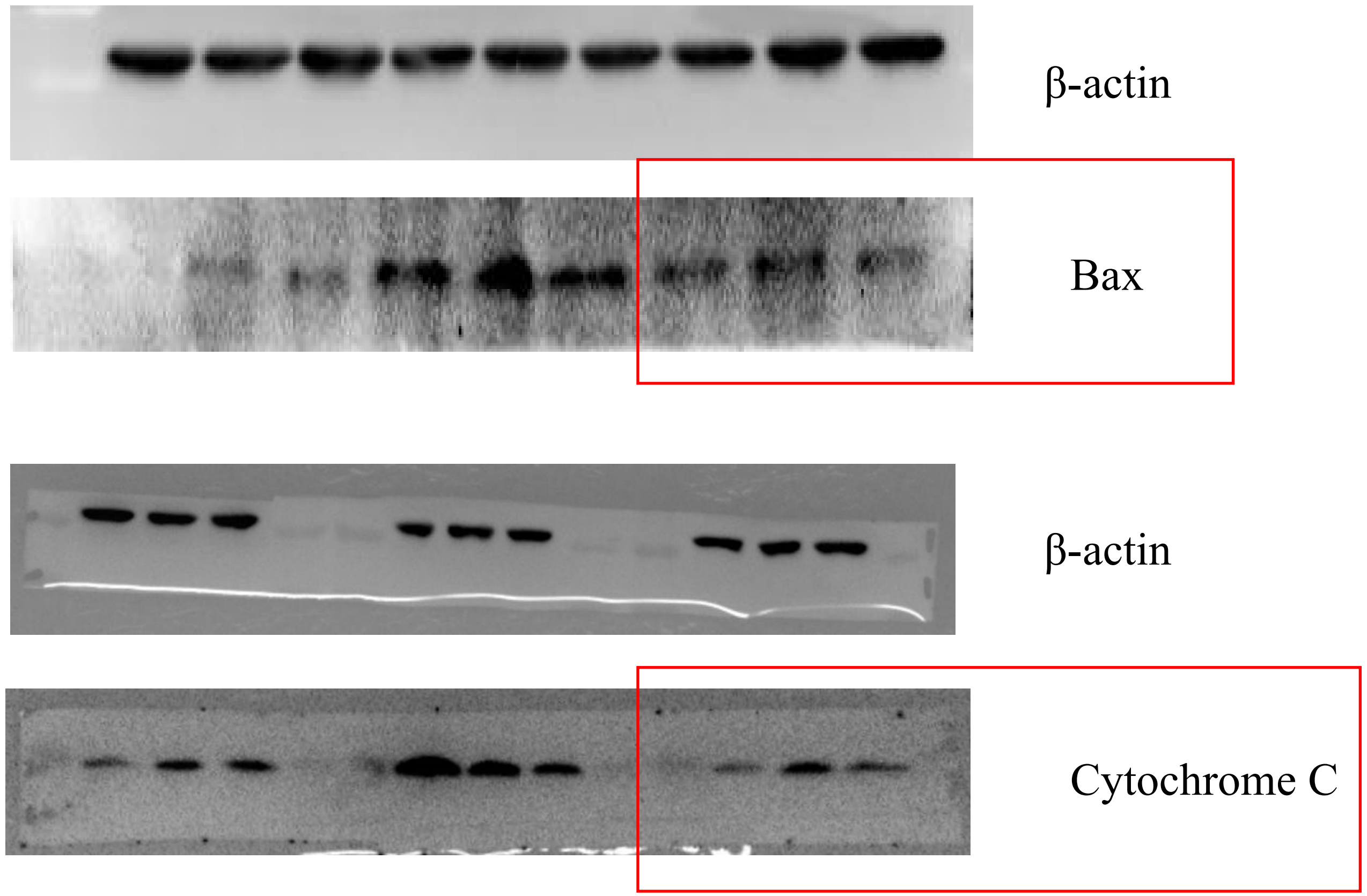
β-actin
Bax
β-actin
Cytochrome C
